# Sex differences in APOE- and PICALM-related cognitive profiles in healthy middle-aged adults

**DOI:** 10.3389/fragi.2025.1694701

**Published:** 2026-01-13

**Authors:** Adam Bednorz, Paulina Trybek, Minh Tuan Hoang, Dorota Religa

**Affiliations:** 1 John Paul II Geriatric Hospital, Katowice, Poland; 2 Institute of Psychology, Humanitas University, Sosnowiec, Poland; 3 Institute of Physics, University of Silesia, Katowice, Poland; 4 Division of Clinical Geriatrics, Department of Neurobiology, Care Sciences and Society, Karolinska Institutet, Stockholm, Sweden; 5 Faculty of Public Health, University of Medicine and Pharmacy, Vietnam National University, Hanoi, Vietnam; 6 Theme Inflammation and Aging, Karolinska University Hospital, Huddinge, Sweden

**Keywords:** Alzheimer’s disease, APOE genotype, PICALM genotype, neuropsychological assessments, machine learning

## Abstract

**Introduction:**

The *APOE* ε4 and *PICALM GG* genotypes are strong genetic risk factors for Alzheimer’s disease. This study aimed to identify cognitive subgroups using unsupervised machine learning and to investigate the influence of *APOE* and *PICALM* genotypes on cognitive performance.

**Material and methods:**

Cognitive, genetic and demographic data from 192 healthy middle-aged adults (50–63 years) from the PEARL-Neuro Database were analyzed using agglomerative hierarchical clustering. Neuropsychological tests included the California Verbal Learning Test, Raven’s Progressive Matrices, and the Edinburgh Handedness Inventory. Subsequent analyses used linear regression models to assess the effects of *APOE*, *PICALM*, and their interaction on cognitive outcomes.

**Results:**

Two cognitive subgroups (better vs. worse performance) were identified for both females (n = 60/43) and males (n = 38/51). In women with lower cognitive performance, the presence of the *APOE* ε3ε4 allele was significantly associated with a higher number of perseverations (CVLT9: $p_{\text{FDR}}=0.02$ , $R^{2}=0.18$ ) and lower recognition accuracy (CVLT12: $p_{\text{FDR}}=0.04$ , $R^{2}=0.12$ ). A significant *PICALM GG* × education interaction was observed for fluid intelligence ( $p_{\text{FDR}}=0.03$ , $R^{2}=0.34$ ). In men with lower cognitive performance, the *APOE* ε3ε4 genotype was associated with lower fluid intelligence scores (RPM: $p_{\text{FDR}}=0.04$ , $R^{2}=0.09$ ). Furthermore, significant *APOE* × *PICALM* interactions were found for verbal learning (CVLT1: $p_{\text{FDR}}=0.03$ , $R^{2}=0.16$ ) as well as delayed cued recall (CVLT6: $p_{\text{FDR}}=0.03$ , $R^{2}=0.12$ ; CVLT8: $p_{\text{FDR}}=0.03$ , $R^{2}=0.13$ ).

**Conclusion:**

This study revealed significant sex differences in gene–cognition interactions. In females with lower cognitive performance, the genotype *APOE* ε3ε4 was associated with poorer recognition, while the combined effects of *APOE* × *PICALM* in males were associated with weaker episodic memory. Although performance remained within normative ranges, these subtle differences may indicate early risk and warrant longitudinal monitoring.

## 1 Introduction

The *APOE* gene, which encodes apolipoprotein E, is a key genetic risk factor for Alzheimer’s disease (AD), with three common alleles: ε 2, ε 3, and ε 4 (Huang et al., 2019; Raulin et al., 2022). The variant ε 4, present in approximately 15% of the population, markedly increases the risk of AD compared to the genotype ε 3/ ε 3 (Huang and Mahley, 2014). Carriers of one ε 4 allele have a three-fold higher risk of late-onset Alzheimer’s disease (LOAD), while homozygotes face up to a 15-fold increase (Hoogmartens et al., 2021; Liu et al., 2013). Although lifetime risk between sexes appears comparable from 55 to 85 years of age, women tend to show greater vulnerability earlier in life, suggesting age-dependent sex effects (Altmann et al., 2014; Neu et al., 2017; Eid et al., 2019). Previous studies suggest that the presence of the *APOE* ε 4 allele is associated with subtle cognitive impairments and an earlier onset of decline in younger populations, with episodic memory deficits potentially associated with hippocampal atrophy observed in healthy older adults and individuals with mild cognitive impairment (MCI) or AD (Lancaster et al., 2016; Sweet et al., 2012; Fan et al., 2019).

The *PICALM* (phosphatidylinositol binding clathrin assembly protein) gene has been identified as a risk locus for late-onset Alzheimer’s disease (LOAD) (Ando et al., 2022; Jansen et al., 2019). The single nucleotide polymorphism (SNP) rs3851179 affects the expression of *PICALM* and correlates with AD biomarkers, including cerebrospinal amyloid- β and tau levels (Xu et al., 2020; Eid et al., 2019). Gene-wide analyses have also identified *PICALM* as a key locus associated with entorhinal cortical thickness, reinforcing its role in AD-related neurodegeneration (Furney et al., 2011). Furthermore, *PICALM* has been implicated in the timing and progression of cognitive decline, with the rs3851179 variant linked to an earlier onset of cognitive deterioration (Sweet et al., 2012). The A-allele is generally considered protective, while the G-allele confers an increased risk of AD (approximately 11%), potentially through altered gene expression and accelerated hippocampal atrophy (Ponomareva et al., 2020; Zeng et al., 2019; Xu et al., 2020). Carriers of the protective A-allele have shown reduced atrophy in the hippocampus, middle temporal gyrus, and the precuneus, as well as faster information processing and a lower risk of cognitive decline (Zeng et al., 2019). In individuals with MCI, the *PICALM* rs3851179 GG genotype has been associated with altered functional connectivity within the default mode network (Sun et al., 2017). Furthermore, rs3851179 has been reported to interact with the disease status to influence gray matter volume and cognition, with carriers of A-alleles exhibiting less putaminal atrophy and better global and executive functions, particularly in early-onset AD (Wu et al., 2022; Wu et al., 2024).

A synergistic adverse effect has also been observed between homozygosity for the risk allele (G) of *PICALM* rs3851179 and the presence of the *APOE* ε 4 allele, in individuals with early AD, resulting in reduced prefrontal volume and poorer performance on the Trail Making Test A, which is sensitive to processing speed (Morgen et al., 2014). Throughout, the association between *PICALM* and AD has been found predominantly in individuals carrying the risk allele *APOE* ε 4, supporting a synergistic interaction between the two genes in modulating the susceptibility of the disease (Jun, 2011). Combined risk genotypes have been associated with reduced episodic memory and a lower cerebral metabolic rate, particularly among carriers of *APOE* ε 4 (Barral et al., 2012; Chang et al., 2019).

The present study aimed to identify separate clusters of cognitive performance in males and females and to assess whether these groups differed in the genetic risk of AD. The *APOE* ε 4 allele has been associated with higher risk in women than in men, although the evidence in the studies remains mixed and not fully consistent (Sampedro et al., 2015; Sundermann et al., 2018). We further examined whether individual differences in neuropsychological performance could be explained by *APOE* ε *4* and *PICALM GG* genotypes, independently and interactively, and whether age and education moderated these associations. Identifying *APOE*–*PICALM* interactions can improve the early detection of individuals at risk of accelerated cognitive decline in late-onset AD (Bellenguez et al., 2022; Barral et al., 2012). Most of the existing evidence for sex-dependent *APOE*–*PICALM* interactions comes from clinical or pathology-enriched samples (MCI/AD). Direct investigations of sex-specific effects of *PICALM* on cognition in middle-aged cognitively healthy adults remain scarce. Although evidence on sex-dependent effects of *PICALM* remains limited, in clinical cohorts, the rs3851179 A-allele has been associated with better cognitive performance and a slower decline in older adults–particularly in men (Mengel-From et al., 2011). However, it remains unclear whether similar sex-dependent effects can be observed prior to the clinical manifestation of cognitive impairment. Therefore, examining the effects related to *APOE*–*PICALM* in cognitively healthy adults provides an opportunity to identify early sex-specific trajectories of cognitive aging.

The study utilized data from the publicly available PEARL-Neuro Database (Dzianok and Kublik, 2024). Previous analyses of this cohort did not find significant demographic or cognitive differences between carriers and non-carriers of *APOE* or *PICALM* risk alleles (Dzianok and Kublik, 2023a). Single *APOE* ε *4* carriers showed slower reaction times under high cognitive load, an effect mitigated by risk variants of *PICALM*, while EEG–fMRI data indicated reduced neural complexity and altered connectivity in at-risk individuals (Dzianok and Kublik, 2023b; Dzianok et al., 2024). To avoid redundancy, the variables that overlapped from these previous analyses were excluded from the present study. Previous Polish studies on *APOE* focused on its association with LOAD, with no additional effects from other loci (Styczynska et al., 2003; Religa et al., 2003).

To uncover latent patterns, we reanalyzed neuropsychological data using unsupervised machine learning, which identifies the structure in unlabeled data without predefined categories (Dalmaijer et al., 2022). Such data-driven approaches–including clustering and dimensionality reduction (e.g., principal component analysis)–are increasingly applied in behavioral genetics and neuropsychology to reveal non-linear associations between cognitive phenotypes and genetic variation (Wang et al., 2024; Scheltens et al., 2016).

## 2 Materials and methods

### 2.1 Dataset and participants

Study participants were selected from the PEARL-Neuro Database. A detailed description of the trial has been described elsewhere (Dzianok and Kublik, 2024). The database contains genetic information on *APOE* and *PICALM* genes. *APOE* (rs429358/rs7412, necessary to identify the main isoforms ε 2, ε 3 and ε 4) and *PICALM* (rs3851179) alleles were genotyped using the traditional Sanger sequencing method, a reliable and well-established DNA sequencing approach (Dzianok and Kublik, 2024). In addition, this database includes psychometric data, basic demographic information, and health data in a group of 192 healthy middle-aged individuals (aged 50–63 years). Of the participants, 77.6% (n = 149) reported higher education, 10.42% (n = 20) had secondary education, and 2.60% (n = 5) had partial higher education, with data missing for 18 individuals. Table 1 presents descriptive statistics for all participants and stratified by sex, while Table 2 summarizes the distribution of *APOE* and *PICALM* genotypes in the male and female groups. These data represent the characteristics of the sample prior to further preprocessing. Only variables assessing cognitive functioning were selected from the dataset, including the California Verbal Learning Test (CVLT), Raven’s Progressive Matrices (RPM), and the Edinburgh Handedness Inventory (EHI), which served as input features for the unsupervised clustering. The definitions of the selected parameters are provided in Table 3.

**TABLE 1:** Descriptive statistics for all participants and stratified by sex.

Variable	All		Female		Male	
	Mean	SD	Mean	SD	Mean	SD
Age	55.052	3.148	54.979	3.228	55.143	3.064
Education	2.740	0.653	2.708	0.679	2.779	0.620
CVLT_1	62.353	8.963	65.240	7.926	58.753	8.924
CVLT_2	9.607	2.016	10.177	1.984	8.896	1.832
CVLT_3	14.000	2.026	14.552	1.589	13.312	2.296
CVLT_4	8.370	1.974	8.792	1.989	7.844	1.836
CVLT_5	12.884	2.674	13.792	2.122	11.753	2.866
CVLT_6	13.520	1.897	14.000	1.473	12.922	2.187
CVLT_7	13.520	2.441	14.281	1.834	12.571	2.765
CVLT_8	13.688	1.978	14.198	1.553	13.052	2.259
CVLT_9	3.954	4.400	4.552	5.066	3.208	3.274
CVLT_10	1.127	1.686	0.990	1.689	1.299	1.679
CVLT_11	0.723	1.556	0.427	0.818	1.091	2.098
CVLT_12	15.428	0.977	15.542	0.767	15.286	1.179
CVLT_13	0.532	1.154	0.396	0.989	0.701	1.319
RPM	52.931	4.532	52.354	5.122	53.649	3.572
EHI	84.464	20.956	83.670	21.395	85.455	20.490

**TABLE 2:** Distribution of APOE and PICALM genotypes by sex.

Genotype	All (n = 192)	Female (n = 103)	Male (n = 89)
APOE 2/2	1	0	1
APOE 2/3	21	12	9
APOE 2/4	3	1	2
APOE 3/3	119	65	54
APOE 3/4	46	25	21
APOE 4/4	2	0	2
PICALM A/G	97	51	46
PICALM A/A	16	8	8
PICALM G/G	79	44	35

**TABLE 3:** Selected cognitive function variables from the dataset.

Variable	Description
CVLT_1	CVLT: List A, trials 1–5 (total learning)
CVLT_2	CVLT: List A, trial 1 (initial recall)
CVLT_3	CVLT: List A, trial 5 (final recall)
CVLT_4	CVLT: List B (interference list)
CVLT_5	CVLT: Short-term delay free recall
CVLT_6	CVLT: Short-term delay cued recall
CVLT_7	CVLT: Long-term delay free recall
CVLT_8	CVLT: Long-term delay cued recall
CVLT_9	CVLT: Perseverations
CVLT_10	CVLT: Intrusion errors – free recall
CVLT_11	CVLT: Intrusion errors – cued recall
CVLT_12	CVLT: Recognition – total hits
CVLT_13	CVLT: Recognition – false alarms
RPM	Raven’s progressive matrices – total score
EHI	Edinburgh Handedness inventory – total score

Abbreviations: CVLT, California verbal learning test; RPM, Raven’s progressive matrices; EHI, Edinburgh handedness inventory.

A description of the methods is given below:

- CVLT - is a widely used tool in clinical and research settings to assess verbal learning and memory. It enables the evaluation of various cognitive processes, including learning between repetitions, serial position effects, semantic clustering, intrusions, and proactive interference. The test was designed with ecological validity, incorporating tasks that reflected daily activities, such as recalling shopping lists (Delis et al., 2008; Elwood, 1995; Łojek et al., 2010).
- RPM - is a nonverbal test designed to assess general cognitive ability, particularly abstract reasoning and problem-solving skills. The research findings indicate that RPM can be considered a relatively independent test of cultural factors to measure fluid intelligence (Raven et al., 2003; Raven, 2008; Jaworowska and Szustrowa, 2010). The allotted time to complete the test was set at 30 min, replacing the unlimited time frame used in the original version (Dzianok and Kublik, 2024).
- EHI - is a brief tool designed to assess handedness on a quantitative scale. This distinction is relevant due to its association with individual differences in neuropsychological and functional brain characteristics (Edlin et al., 2015).

### 2.2 Machine learning approach and statistical analysis

**FIGURE 1:**
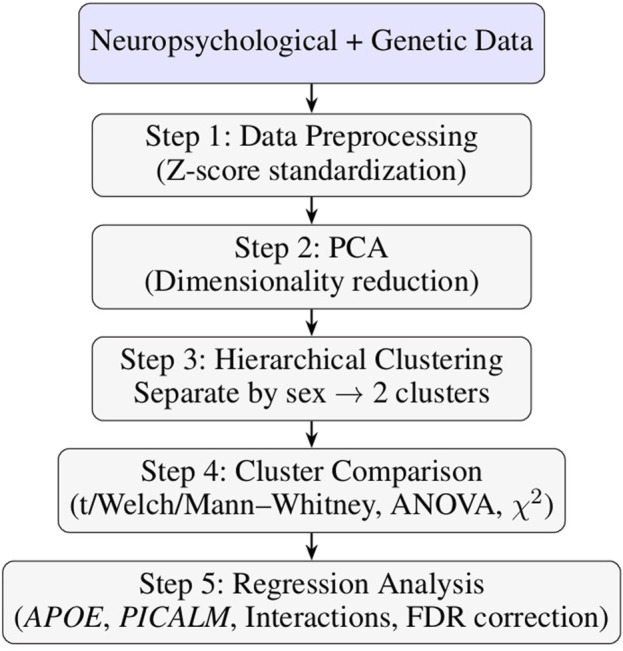
Overview of the machine learning and statistical analysis workflow.

The diagram below presents an overview of the analytical workflow used in this study (see Figure 1).

- Step 1: The dataset was comprehensively pre-processed statistically. In the initial step, z-score standardization was applied to normalize the scale of the variables and ensure their comparability. Each feature was transformed to have a mean of zero and a standard deviation of one, which was essential due to the use of the Euclidean distance metric in subsequent analyses.
- Step 2: Principal Component Analysis (PCA) was used to reduce dimensionality and identify the main sources of variance within the dataset (Greenacre et al., 2022). The first two principal components were used to illustrate the clustering results.
- Step 3: Agglomerative hierarchical clustering was applied to explore the latent structure within the neuropsychological dataset. This unsupervised learning method is well suited to small and moderately sized datasets typical of clinical research, where group labels are often unknown or hypothetical (Li et al., 2022). Clustering was conducted using the AgglomerativeClustering algorithm from the scikit-learn library with Euclidean distance and Ward’s linkage, which minimizes within-cluster variance and is appropriate for continuous cognitive data. To compute the distance between observations, the standard Euclidean distance was used, defined as:

$$d\left(x,y\right)=\sqrt{\overset{n}{\underset{i=1}{\sum }}{\left(x_{i}-y_{i}\right)}^{2}}$$

where x and y are two observations in n -dimensional space.

Agglomerative clustering has been shown to be useful in genotype-to-phenotype studies (Sasirekha and Baby, 2013). Although this analysis originally aimed to cluster based on genotype, the limited number of rare variants (e.g., individuals carrying two ε4 alleles) precluded meaningful comparisons. Therefore, a reverse approach was adopted–clustering based on cognitive performance (phenotype) and subsequently examining the distribution of *APOE* and *PICALM* genotypes within clusters. The ambiguous genotype *APOE* ε2ε4 was excluded from further analyses. The number of clusters was estimated based on dendrogram inspection and by maximizing the silhouette score, a metric that evaluates how well each observation fits within its assigned cluster compared to others. The hierarchical dendrogram was cut at a distance level of approximately 26.161 for males and 29.904 for females, yielding two clusters determined by the parameter n_clusters = 2 in the Agglomerative Clustering model. Hierarchical clustering was performed separately for males and females to account for established sex-specific patterns in cognitive performance and APOE/PICALM effects, thus avoiding potential bias or dilution of within-sex structure that might arise if sex were treated only as a covariate. To assess whether the sample sizes obtained through clustering were adequate, an *a priori* power analysis was conducted. Assuming α=0.05 , power = 0.80, and medium-to-large effect size (Cohen’s *d* = 0.6), the estimated minimum sample size per group was approximately 45 participants, totaling 89 for balanced groups (Brydges, 2019; Lakens, 2022). In our study, the resulting clusters comprised 43 and 60 women, and 38 and 51 men, that align with methodological recommendations for unsupervised learning approaches, which suggest a minimum of 20–30 participants per subgroup (Dalmaijer et al., 2022).

- Step 4: To assess differences between clusters, cognitive variables were compared using parametric or nonparametric tests, depending on data distribution (Shapiro–Wilk) and variance homogeneity (Levene’s test). Student’s or Welch’s t-tests were applied for normally distributed data, and the Mann–Whitney U test for non-normal distributions. Differences in genotype frequencies were examined using chi-square or Fisher’s exact tests. Mean neuropsychological scores across the four clusters (two female, two male) were compared using ANOVA with Tukey’s *post hoc* tests to identify significant differences between-group.
- Step 5 Linear regression analyses were conducted within each cluster to assess the effects of genetic risk genotypes–*APOE* (ε3ε4) and *PICALM* GG–on neuropsychological performance. Three main models for each cognitive variable were estimated: including *APOE*, *PICALM*, and their interaction (*APOE* × *PICALM*). Additional models examined interactions with age and education, including higher-order terms (e.g., *APOE* × age × education). For each model, regression coefficients (β) , p-values and coefficients of determination $\left(R^{2}\right)$ were recorded. To account for multiple comparisons, all p-values obtained from the regression analyses were adjusted using the False Discovery Rate (FDR) correction according to the Benjamini–Hochberg procedure (Benjamini and Hochberg, 1995). The FDR-adjusted p-values $\left(p_{\mathrm{FDR}}\right)$ were calculated separately for the better- and worse-performing cognitive clusters. Only effects with $p_{\mathrm{FDR}}<0.05$ were considered statistically significant. The analyses were performed separately for the cognitively “better” and “worse” clusters, which reflect relative cognitive profiles rather than clinical categories. Clustering was performed in the entire neuropsychological dataset to preserve the structure of the data-driven group, while regression analyses were limited to carriers of single risk alleles (*APOE* ε 3/ ε 4 and *PICALM* GG) to ensure consistency between sexes. All analyses were performed in Python 3.0; the code is available on request.

## 3 Results

### 3.1 Clustering analysis for female and male participants

**FIGURE 2:**
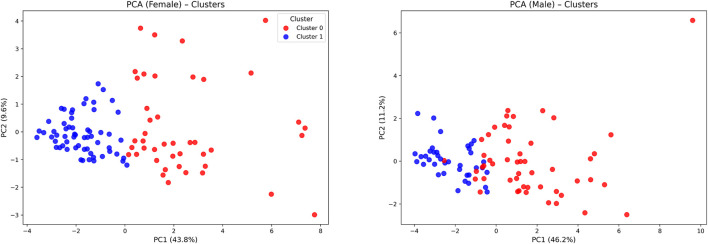
Clustering based on principal component analysis (PCA) in female (left panel) and male (right panel) groups, illustrating group separation in a two-dimensional space.

**FIGURE 3:**
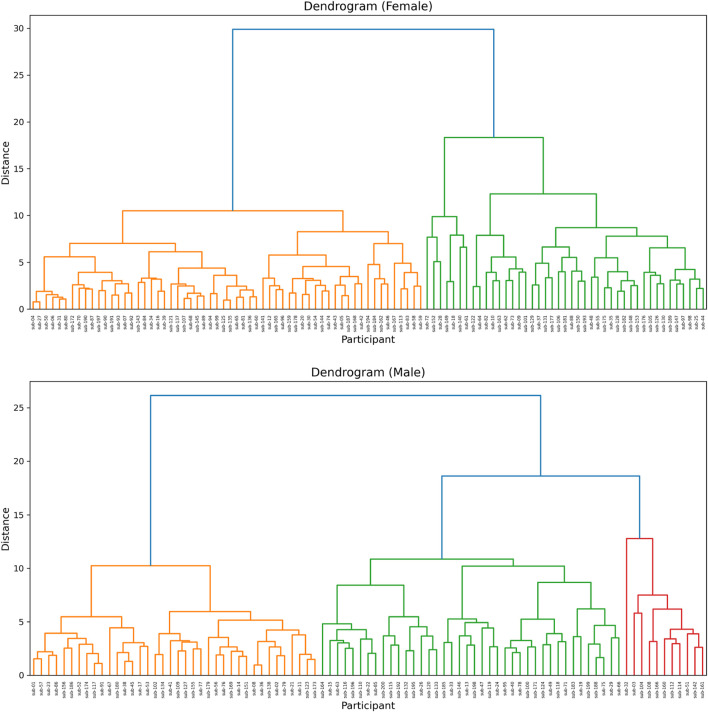
Hierarchical clustering dendrograms obtained using Ward’s method for the female (upper panel) and male (lower panel) groups. Each data point starts as an individual cluster, and clusters are progressively merged based on similarity.

The cluster analysis conducted separately for female and male participants revealed two distinct groups within each sex. Visualization using principal component analysis (PCA) showed clear separation between clusters in both females and males (Figure 2). Hierarchical clustering further supported the presence of two main clusters within each sex (Figure 3). No significant differences were observed between clusters in age or education (Table 4). As expected, since the clustering was based on measures of neuropsychological performance, all neuropsychological variables differed significantly between the clusters (p<0.05) .Therefore, these findings serve primarily to characterize the cognitive profiles of the newly identified clusters in both female and male. In both groups, the participants in Cluster 1 consistently outperformed those in Cluster 0 in all CVLT measures. This included higher total learning scores across trials (CVLT_1), better performance in both initial and final learning trials (CVLT_2–CVLT_3), and superior results in delayed recall conditions–covering short-delay free recall and long-delay free and cued recall tasks (CVLT_5–CVLT_8). Individuals in Cluster 1 also made fewer errors overall, including perseveration errors (CVLT_9) and intrusion errors in both free (CVLT_10) and cued recall (CVLT_11). Recognition performance also favored Cluster 1, reflected in a higher number of correct hits (CVLT_12) and fewer false alarms (CVLT_13). Beyond verbal memory, participants in Cluster 1 also demonstrated higher fluid intelligence scores (RPM) and a higher degree of right-handedness as measured by EHI.

**TABLE 4:** Characteristics of participants and genotype distribution by cognitive cluster in female and male groups together.

Measure	Female			Male		
	Cluster 0 (n = 43)	Cluster 1 (n = 60)	p-value	Cluster 0 (n = 51)	Cluster 1 (n = 38)	p-value
Age	55.30 (3.39)	54.67 (3.05)	​	54.98 (2.69)	55.47 (3.31)	​
Education	2.65 (0.74)	2.75 (0.63)	​	2.72 (0.70)	2.85 (0.50)	​
*APOE* genotype						
ε 2/ ε 2	—	—	​	0	1	​
ε 2/ ε 3	7	5	​	4	5	​
ε 2/ ε 4	0	1	​	2	0	​
ε 3/ ε 3	26	39	​	34	20	​
ε 3/ ε 4	10	15	​	10	11	​
ε 4/ ε 4	—	—	​	1	1	​
*PICALM* genotype						
A/G	24	27	​	28	18	​
A/A	2	6	​	4	4	​
G/G	17	27	​	19	16	​
CVLT_1	58.256 ± 7.004	69.583 ± 4.681	***	52.510 ± 7.223	66.158 ± 5.175	***
CVLT_2	9.023 ± 1.779	10.833 ± 1.729	***	8.137 ± 1.600	9.789 ± 1.877	***
CVLT_3	13.326 ± 1.507	15.333 ± 0.951	***	11.843 ± 2.072	15.000 ± 1.252	***
CVLT_4	8.140 ± 1.910	9.117 ± 1.992	*	7.176 ± 1.763	8.500 ± 1.900	**
CVLT_5	11.860 ± 1.934	15.033 ± 0.938	***	10.098 ± 2.579	13.711 ± 1.707	***
CVLT_6	12.581 ± 1.159	14.983 ± 0.770	***	11.549 ± 2.052	14.500 ± 1.084	***
CVLT_7	12.674 ± 1.782	15.383 ± 0.715	***	10.706 ± 2.540	14.789 ± 1.018	***
CVLT_8	12.814 ± 1.402	15.167 ± 0.763	***	11.784 ± 2.230	14.632 ± 1.125	***
CVLT_9	6.209 ± 5.621	3.533 ± 4.451	*	4.725 ± 4.000	1.763 ± 1.895	***
CVLT_10	1.605 ± 2.227	0.517 ± 0.833	**	1.803 ± 1.833	0.680 ± 0.990	***
CVLT_11	0.740 ± 1.049	0.200 ± 0.480	**	1.549 ± 2.436	0.553 ± 1.032	*
CVLT_12	15.071 ± 0.921	15.883 ± 0.324	***	14.725 ± 1.537	15.789 ± 0.474	***
CVLT_13	0.881 ± 1.347	0.003 ± 0.181	***	1.098 ± 1.473	0.132 ± 0.414	***
RPM	50.698 ± 4.983	53.433 ± 4.731	**	51.961 ± 3.934	55.263 ± 2.947	***
EHI	74.108 ± 26.754	89.650 ± 14.505	**	81.830 ± 23.241	89.474 ± 16.431	​
Fisher/ $\chi^{2}$ test for APOE genotype						​
Fisher/ $\chi^{2}$ test for PICALM genotype						​

Abbreviations: CVLT, California verbal learning test; RPM, Raven’s progressive matrices, EHI, Edinburgh handedness inventory.

Significance levels: *p < 0.05, **p < 0.01, ***p < 0.001.

In all cognitive measures, one-way ANOVA tests revealed highly significant group effects (all p < 0.001), with *post hoc* Tukey comparisons indicating that the “female better” cluster consistently outperformed the other groups on nearly all CVLT variables, similar patterns were observed for the RPM and EHI measures, although the differences between the higher-performing clusters were not statistically significant (see Supplementary Materia1 1.2) Additional boxplots and histograms illustrating the distributions of individual neuropsychological measures (CVLT_1–CVLT_13, RPM, and EHI) for each subgroup are provided in the Supplementary Material 1.3 and 1.5 to facilitate the evaluation of within-group variability and possible ceiling or floor effects.

**FIGURE 4:**
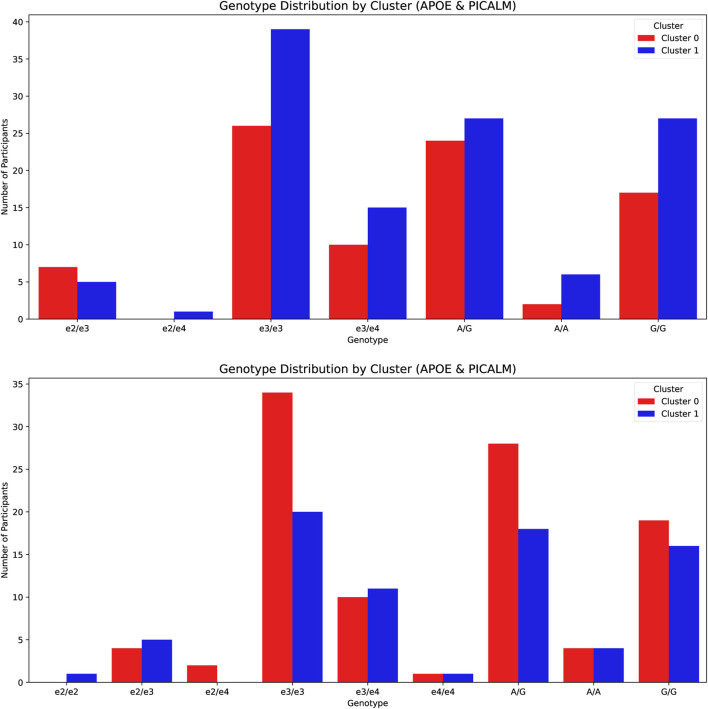
Distribution of APOE and PICALM genotypes by cluster for the female (upper panel) and male (lower panel) groups. Genotype counts are shown separately for the cognitive “better” and “worse” clusters within each sex.

Fisher’s exact test revealed no statistically significant differences in genotype distribution in clusters in either sex (*APOE*: p=0.68 for females, p=0.41 for males; *PICALM*: p=0.53 for females, p=0.76 for males). This indicates that cluster membership was not significantly associated with the *APOE* or *PICALM* genotypes in the present sample. Detailed results are presented in Table 4 and illustrated in Figure 4 (upper and lower panels for females and males, respectively).

To account for potential somatic influences on cognitive performance, additional analyses of basic clinical indicators were conducted; however, blood count data were available for only a subset of participants, substantially limiting interpretability (see Supplementary Material 1.4).

### 3.2 Genetic influence of APOE and PICALM and their interaction on cognitive performance - in female’s group

#### 3.2.1 Worse-performing cognitive cluster

Only the results that survived the Benjamini–Hochberg false discovery rate (FDR) correction $\left(p_{\text{FDR}}<0.05\right)$ are reported in this section; non-significant associations ( p<0.05 but $p_{\text{FDR}}>0.05$ ) were omitted for clarity. The percentages reported in parentheses refer to the proportion of explained variance $\left(R^{2}\right)$ for each model, as shown in Table 5. All other interactions are reported in the Supplementary Material 1.1.

**TABLE 5:** Regression models for APOE and PICALM effects in females: comparison between better (blue) and worse (red) cognitive clusters.

Variable	$\beta_{\text{better}}$	95% CI	$p_{\text{better}}$	$pFDR_{\text{better}}$	${\text{R}}_{\text{better}}^{2}$	$\beta_{\text{worse}}$	95% CI	$p_{\text{worse}}$	$pFDR_{\text{worse}}$	${\text{R}}_{\text{worse}}^{2}$
Model 1: APOE_e3e4										
CVLT_9	−2.044	[−4.669, 0.580]	0.124	​	0.040	5.591	[1.837, 9.345]	0.004	*	0.181
CVLT_12	−0.022	[−0.217, 0.173]	0.820	​	0.001	−0.798	[−1.459, −0.137]	0.019	*	0.129
Model 1a: APOE_e3e4 × age										
CVLT_5	−0.236	[−0.419, −0.052]	0.013	​	0.116	0.137	[−0.384, 0.657]	0.598	​	0.124
CVLT_12	0.008	[−0.059, 0.074]	0.809	​	0.018	−0.386	[−0.679, −0.092]	0.011	*	0.276
CVLT_13	0.023	[−0.013, 0.060]	0.209	​	0.004	−0.534	[−1.004, −0.066]	0.027	​	0.138
Model 1b: APOE_e3e4 × education										
CVLT_4	−1.677	[−3.875, 0.521]	0.132	​	0.043	2.408	[0.311, 4.505]	0.026	​	0.132
CVLT_13	0.020	[−0.195, 0.236]	0.850	​	0.015	−2.170	[−3.601, −0.741]	0.004	*	0.228
Model 1c: APOE_e3e4 × age × education										
CVLT_5	−0.065	[−0.125, −0.006]	0.031	​	0.160	−0.436	[−1.910, 1.047]	0.553	​	0.272
CVLT_9	0.018	[−0.283, 0.320]	0.903	​	0.094	−4.063	[−7.261, −0.866]	0.014	*	0.550
CVLT_12	0.0004	[−0.021, 0.022]	0.969	​	0.179	−0.590	[−1.136, −0.043]	0.035	​	0.583
Model 2: PICALM_GG										
CVLT_10	−0.131	[−0.567, 0.304]	0.548	​	0.006	−2.167	[−3.412, −0.923]	0.001	*	0.231
CVLT_11	−0.094	[−0.344, 0.156]	0.454	​	0.010	−0.841	[−1.455, −0.228]	0.008	*	0.158
Model 2a: PICALM_GG × age										
CVLT_2	−0.328	[−0.627, −0.029]	0.032	​	0.101	−0.235	[−0.585, 0.116]	0.184	​	0.068
CVLT_5	−0.185	[−0.347, −0.022]	0.027	​	0.101	−0.035	[−0.417, 0.347]	0.852	​	0.065
Model 2b: PICALM_GG × education										
RPM	−1.023	[−5.506, 3.460]	0.649	​	0.011	−5.366	[−9.414, −1.319]	0.011	*	0.347
Model 2c: PICALM_GG × age × education										
CVLT_4	−0.283	[−3.104, 2.539]	0.841	​	0.086	0.563	[0.033, 1.094]	0.038	​	0.208
Model 3: APOE × PICALM										
CVLT_10	1.200	[0.217, 2.184]	0.018	​	0.102	−0.057	[−3.042, 2.927]	0.969	​	0.232
CVLT_11	0.786	[0.229, 1.343]	0.007	​	0.133	−0.698	[−2.137, 0.742]	0.333	​	0.195
Model 3a: APOE × PICALM × age										
CVLT_8	0.038	[−0.304, 0.379]	0.826	​	0.061	1.081	[0.224, 1.938]	0.015	*	0.225
Model 3b: APOE × PICALM × education										
CVLT_10	0.359	[0.036, 0.681]	0.030	​	0.116	−0.198	[−1.245, 0.848]	0.702	​	0.301
CVLT_11	0.216	[0.035, 0.398]	0.021	​	0.167	−0.231	[−0.749, 0.287]	0.371	​	0.208
Model 3c: APOE × PICALM × age × education										
CVLT_5	−0.033	[−0.156, 0.090]	0.587	​	0.269	0.539	[0.143, 0.935]	0.010	*	0.503
CVLT_6	−0.023	[−0.130, 0.083]	0.664	​	0.173	0.286	[0.021, 0.551]	0.037	​	0.367
CVLT_7	0.006	[−0.098, 0.110]	0.910	​	0.139	0.428	[0.039, 0.817]	0.033	​	0.442
CVLT_8	0.018	[−0.094, 0.129]	0.752	​	0.103	0.517	[0.232, 0.802]	0.001	*	0.514
EHI	−0.803	[−2.837, 1.232]	0.431	​	0.155	6.020	[0.285, 11.755]	0.040	​	0.447

Abbreviations: CVLT, California Verbal Learning Test; RPM, Raven’s Progressive Matrices, EHI, Edinburgh Handedness Inventory.

* pFDR <0.05, ** pFDR <0.01, *** pFDR <0.001.

The presence of the *APOE* ε3ε4 allele was associated with a higher number of perseverations (CVLT_9) and lower recognition performance (CVLT_12), with the genetic factor explaining approximately 18% and 13% of the variance in neuropsychological test performance, respectively. Significant *APOE* × age interactions were observed for recognition performance (CVLT_12), and an *APOE* × education interaction remained significant for false recognition (CVLT_13), with the respective models explaining approximately 28% and 23% of the variance (see Table 5). The *PICALM GG* genotype was significantly associated with a lower number of intrusion errors (CVLT_10; $R^{2}=0.23$ , CVLT_11; $R^{2}=0.16$ ). Additionally, a significant *PICALM* × education interaction was observed for fluid intelligence (RPM), which explained approximately 35% of the variance. Finally, a three-way interaction of *APOE* × *PICALM* × age was detected for long-delay cued recall (CVLT_8), explaining about 22% of variance. Furthermore, a significant four-way interaction of *APOE* × *PICALM* × age × education was detected for CVLT_5 (short-delay free recall; 50% of variance explained) and CVLT_8 (long-delay cued recall; 51% of variance explained) (see Table 5).

#### 3.2.2 Better-performing cognitive cluster

Several nominally significant associations were initially observed in the better-performing cognitive cluster, including interactions involving *APOE*, *PICALM*, age, and education. However, after applying the FDR (Benjamini–Hochberg) correction for multiple comparisons, none of these effects remained statistically significant ( $p_{\mathrm{FDR}}$ > 0.05). Data are presented in Table 5.

### 3.3 Genetic influence of APOE and PICALM and their interaction on cognitive performance - in male’s group

#### 3.3.1 Worse-performing cognitive cluster

In this cluster, the *APOE* ε 4 allele was negatively associated with fluid intelligence (RPM), explaining approximately 10% of the variance, whereas the interaction with education increased the explained variance to around 16% (see Table 6). In the three-way *APOE* × age × education analysis, significant effects were observed for several CVLT measures related to short- and long-delay recall (CVLT_5–CVLT_8), with models that explained between 27% and 36% of variance. For the *PICALM* gene, no significant main effects were observed; however, interactions with education accounted for approximately 13% of the variance in false recognition errors (CVLT_13) and perseveration errors (CVLT_9), respectively. Significant *APOE* × *PICALM* interactions were observed in several memory measures (CVLT_1, CVLT_3, CVLT_6, CVLT_8, CVLT_13), with models explaining between 13% and 23% of the variance (see Table 6). Furthermore, significant three-way *APOE* × *PICALM* × age interactions were found for CVLT_11 and CVLT_13, with explained variance ranging from 52% to 58%. Similarly, *APOE* × *PICALM* × education interactions were observed for CVLT_5–CVLT_8, representing 25%–32% of the variance in recall performance. Finally, a significant four-way interaction *APOE* × *PICALM* × age × education was found for CVLT_11 (intrusion errors), which explained approximately 57% of the variance (see Table 6).

**TABLE 6:** Regression models for APOE and PICALM effects in males: comparison between better (blue) and worse (red) cognitive clusters.

Variable	$\beta_{\text{better}}$	95% CI	$p_{\text{better}}$	pFD ${\text{R}}_{\text{better}}$	${\text{R}}_{\text{better}}^{2}$	$\beta_{\text{worse}}$	95% CI	$p_{\text{worse}}$	pFD ${\text{R}}_{\text{worse}}$	${\text{R}}_{\text{worse}}^{2}$
Model 1: APOE_e3e4										
RPM	0.525	[−1.635, 2.685]	0.625	​	0.007	−3.061	[−5.737, −0.385]	0.026	*	0.097
Model 1a: APOE_e3e4 × age										
CVLT_4	0.494	[0.068, 0.920]	0.024	​	0.143	0.108	[−0.309, 0.524]	0.606	​	0.076
CVLT_5	0.401	[0.015, 0.786]	0.042	​	0.131	−0.468	[−1.082, 0.146]	0.132	​	0.060
Model 1b: APOE_e3e4 × education										
CVLT_9	−2.736	[−5.393, −0.077]	0.044	​	0.144	−3.260	[−6.830, 0.309]	0.072	​	0.092
RPM	1.190	[−3.135, 5.515]	0.578	​	0.088	−4.128	[−7.413, −0.842]	0.015	*	0.159
Model 1c: APOE_e3e4 × age × education										
CVLT_5	0.108	[−0.013, 0.229]	0.077	​	0.277	−1.159	[−2.021, −0.297]	0.010	*	0.273
CVLT_6	−0.002	[−0.094, 0.090]	0.967	​	0.182	−0.872	[−1.521, −0.222]	0.010	*	0.270
CVLT_7	0.029	[−0.055, 0.114]	0.487	​	0.213	−1.131	[−1.868, −0.394]	0.004	*	0.364
CVLT_8	0.006	[−0.095, 0.107]	0.904	​	0.183	−0.889	[−1.567, −0.211]	0.012	*	0.361
CVLT_9	−0.118	[−0.276, 0.040]	0.136	​	0.223	−1.724	[−2.917, −0.530]	0.006	*	0.343
CVLT_13	−0.014	[−0.047, 0.019]	0.382	​	0.159	0.585	[0.057, 1.113]	0.031	​	0.305
Model 2a: PICALM_GG × age										
CVLT_3	0.045	[−0.220, 0.310]	0.731	​	0.016	−0.461	[−0.886, −0.037]	0.034	​	0.131
CVLT_13	0.084	[0.008, 0.159]	0.031	​	0.270	0.217	[−0.085, 0.519]	0.155	​	0.128
Model 2b: PICALM_GG × education										
CVLT_9	1.000	[−1.740, 3.740]	0.462	​	0.069	3.670	[0.502, 6.837]	0.024	*	0.129
Model 2c: PICALM_GG × age × education										
EHI	−33.901	[−52.427, −15.375]	0.001	*	0.426	8.242	[−12.485, 28.970]	0.426	​	0.051
Model 3: APOE × PICALM										
CVLT_1	−5.036	[−12.865, 2.794]	0.200	​	0.062	−13.431	[−23.354, −3.507]	0.009	*	0.160
CVLT_3	−0.210	[−2.162, 1.743]	0.829	​	0.004	3.430	[−6.276, −0.583]	0.019	*	0.160
CVLT_5	1.731	[−0.866, 4.328]	0.185	​	0.051	−3.791	[−7.454, −0.129]	0.043	​	0.102
CVLT_6	0.588	[−1.090, 2.267]	0.481	​	0.018	−3.601	[−6.476, −0.726]	0.015	*	0.127
CVLT_8	0.026	[−1.706, 1.759]	0.976	​	0.029	−3.990	[−7.107, −0.872]	0.013	*	0.130
CVLT_10	1.574	[0.140, 3.008]	0.032	​	0.139	−0.185	[−2.909, 2.540]	0.892	​	0.017
CVLT_11	−0.660	[−2.208, 0.889]	0.393	​	0.077	3.508	[0.109, 6.907]	0.043	​	0.133
CVLT_13	−0.326	[−0.957, 0.305]	0.301	​	0.047	2.928	[0.997, 4.860]	0.004	*	0.235
Model 3a: APOE × PICALM × age										
CVLT_2	−0.369	[−1.295, 0.558]	0.423	​	0.120	−0.804	[−1.553, −0.057]	0.036	​	0.219
CVLT_8	0.054	[−0.527, 0.635]	0.851	​	0.039	−0.994	[−1.910, −0.077]	0.034	​	0.396
CVLT_11	−0.415	[−0.882, 0.053]	0.080	​	0.260	1.666	[0.779, 2.553]	0.000	*	0.526
CVLT_13	−0.187	[−0.354, −0.021]	0.029	​	0.416	0.855	[0.350, 1.360]	0.001	*	0.580
Model 3b: APOE × PICALM × education										
CVLT_5	0.631	[−0.072, 1.334]	0.077	​	0.376	5.583	[0.854, 10.313]	0.022	*	0.249
CVLT_6	0.210	[−0.359, 0.779]	0.456	​	0.195	3.899	[0.344, 7.455]	0.033	​	0.250
CVLT_7	0.059	[−0.456, 0.573]	0.817	​	0.254	5.640	[1.394, 9.887]	0.011	*	0.275
CVLT_8	0.162	[−0.464, 0.788]	0.600	​	0.190	4.329	[0.568, 8.090]	0.025	*	0.325
Model 3c: APOE × PICALM × age × education										
CVLT_10	0.171	[0.008, 0.334]	0.041	​	0.327	0.284	[−0.061, 0.628]	0.103	​	0.247
CVLT_11	−0.035	[−0.151, 0.080]	0.533	​	0.196	0.501	[0.150, 0.851]	0.007	*	0.572
EHI	2.314	[0.527, 4.101]	0.013	​	0.707	0.324	[−4.084, 4.732]	0.881	​	0.130

Abbreviations: CVLT, California verbal learning test; RPM, Raven’s progressive matrices; EHI, Edinburgh handedness inventory.

* pFDR <0.05, ** pFDR <0.01, *** pFDR <0.001.

#### 3.3.2 Better-performing cognitive cluster

In this cluster, a significant three-way interaction (*PICALM* GG × age × education) was observed only for handedness $\left(R^{2}=0.43\right)$ . Although several nominal associations were observed before correction (e.g., CVLT_4, CVLT_5), none remained a significant adjustment of the FDR (all $p_{\mathrm{FDR}}$ > 0.05). Data are presented in Table 6.

## 4 Discussion

### 4.1 Cognitive profiles and clustering patterns in female group’s

Within the lower-performing female cluster, carriers of the genotype *APOE* ε3ε 4 showed significantly lower recognition precision and a higher number of perseverative errors. No such associations were observed in the higher-performing group. The *APOE* ε 4 allele has been consistently associated with recognition difficulties in both clinical and healthy groups, including a faster decline in word recognition and overall poorer recognition performance (Hirono et al., 2003; Haley et al., 2010). Perseverations, commonly observed in AD and list-learning tasks, are believed to reflect deficits in working memory and response monitoring (Pakhomov et al., 2018; Miozzo et al., 2013), consistent with reports of reduced working memory in cognitively healthy carriers ε 4, particularly under interference-control demands (Reinvang et al., 2010). However, given that the participants were cognitively intact, a certain level of perseverations remains within normative limits (Łojek et al., 2010), especially with extended word lists. Thus, these findings should be interpreted with caution. Although the *PICALM* GG genotype is considered a risk variant, our findings–showing fewer intrusion errors among GG carriers–may reflect delayed expression of its adverse effects or the influence of cognitive reserve (Pinto and Tandel, 2016). In line with previous reports indicating that cognitive reserve can mitigate the detrimental impact of the *APOE* ε 4 allele on memory performance (Pettigrew et al., 2013), a similar compensatory mechanism may also operate in the case of the *PICALM* GG genotype. Alternatively, a form of antagonistic pleiotropy, similar to that proposed for the *APOE* ε *4* allele (Fan et al., 2019; Tuminello and Han, 2011; Rusted et al., 2013), cannot be excluded and warrant further investigation.

### 4.2 Cognitive profiles and clustering patterns in male group’s

The presence of the *APOE* ε 3/ ε 4 genotype was associated with lower performance on the RPM task, reflecting reduced fluid intelligence. Our findings indicate that the *APOE* × *PICALM* interaction significantly affects multiple stages of verbal learning and episodic memory, suggesting that *PICALM* may modulate *APOE*-related cognitive vulnerability (Morgen et al., 2014), and in the lower-performing male cluster this pattern resembles a “flip-flop effect,” where the direction of genetic influence depends on the allelic context of another gene (Engelman et al., 2013; Liu et al., 2023; Fan et al., 2019). However, since cognitively healthy male carriers ε 4 also show poorer episodic memory (Sundermann et al., 2018), it is not clear whether the observed deficits reflect the combined effect of *APOE* × *PICALM* or *APOE* ε 4 alone. Consistent with previous work, carriers of *APOE* ε *4* exhibit reduced performance in episodic memory, executive functions, and nonverbal cognition, and *APOE* interacts with early-life cognitive ability to influence processing speed and variability of reaction time in older adults (Schultz et al., 2008; Liu et al., 2013; Izaks et al., 2011; Luciano et al., 2009). Longitudinal studies are needed to clarify these relationships.

In the lower-performing male cluster, carriers of the *APOE* ε 4 and *PICALM* GG genotypes showed increased intrusion errors (CVLT_11) and false recognitions (CVLT_13), but only in interaction models including age and education (*APOE* × *PICALM* × age; *APOE* × *PICALM* × age × education). Older age combined with these risk genotypes was associated with increased intrusions and false recognitions, reflecting a greater susceptibility to memory interference and reduced retrieval accuracy. This pattern may reflect subtle deficits in inhibitory control or memory monitoring, consistent with prior evidence linking intrusion errors in verbal memory tasks to an increased risk of progression from normal cognition to MCI and early dementia (Thomas et al., 2018; Torres et al., 2019). Age can modulate the cognitive impact of the *APOE* ε *4* allele, with younger carriers showing relatively preserved recall and older adults showing decline (Fan et al., 2019; Caselli et al., 2009; Jochemsen et al., 2012), although results remain mixed (Bunce et al., 2014).

### 4.3 Sex-specific genetic interactions and cognitive performance

Analysis of *APOE-PICALM* interactions revealed different sex-dependent patterns, with males with cognitively lower performance showing deficits related to *APOE* ε 4 in fluid intelligence, and combined effects of *APOE-PICALM* on episodic memory. In females, the associations mainly involved *APOE*-related influences on recognition and executive-control measures, including increased perseverative errors among ε 4 carriers. Although our study examined young, cognitively healthy adults, the sex-dependent *APOE–PICALM* patterns may represent early, subclinical manifestations of cognitive vulnerabilities that only later become clinically apparent. These findings are consistent with previous evidence that the detrimental effect of the *APOE* ε 4 allele on verbal memory is primarily evident in cognitively normal men, while women tend to maintain better memory performance despite a comparable amyloid burden (Sundermann et al., 2018). This pattern may reflect enhanced compensatory mechanisms in women, which could delay the manifestation of cognitive deficits related to *APOE* ε 4 until more advanced stages of the disease (Sundermann et al., 2018; Jack Jr et al., 2013). In general, males more often exhibited higher-order effects (e.g., gene × age × education) with greater explanatory power, suggesting that cognitive performance in men may depend on a more complex gene–environment interplay, but such sex-dependent interaction patterns may help explain part of the missing heritability and variability in cognitive aging trajectories (Singhal et al., 2023; Altmann et al., 2014; Neu et al., 2017; Sundermann et al., 2018). In both lower-performing clusters, education emerged as a key modifier of genetic effects. Among females, a significant *PICALM_GG* × education interaction was found for fluid intelligence (RPM), suggesting that higher educational attainment mitigated the negative impact of the *PICALM* GG risk genotype, possibly reflecting a compensatory role of cognitive reserve (Pinto and Tandel, 2016). In males, the *APOE* ε 4 allele was negatively associated with fluid intelligence, with education increasing the explained variance from approximately 10%–16%. Furthermore, *APOE* × *PICALM* × education interaction was observed for short- and long-term recall (CVLT_5–8), indicating that higher education buffered the adverse cognitive effects of genetic risk. This pattern aligns with evidence that education and related aspects of cognitive reserve can moderate the impact of genetic vulnerability on memory and overall cognitive functioning in older adults (Hsu et al., 2025; Walsemann et al., 2025; Pettigrew et al., 2023; Eid et al., 2019). Similar gene–education interactions have also been reported in AD cohorts, where the cognitive and neural effects of these loci are influenced by age, disease stage, and cognitive reserve (Morgen et al., 2014; Chang et al., 2021; Vonk et al., 2019; Liu et al., 2023; Fan et al., 2019). The relatively high educational level of the current sample may therefore have reduced between-group variability. With greater diversity in educational attainment, cognitive score distributions might have differed, as genetic factors explain more variance in individuals with lower education. Previous research shows that higher education and other cognitive-reserve proxies support better cognitive performance, helping to maintain functioning despite aging or pathology (Le Carret et al., 2003; Panico et al., 2023). It should be noted that educational attainment, although often treated as environmental, is partly heritable (40%–60%) and a recognized modifiable risk factor for dementia (Ayorech et al., 2017; Livingston et al., 2024). The main effects of handedness were not found in worse-performing clusters, consistent with previous large-scale studies that did not show a relationship between handedness and the genetic risk of AD (Hubacek et al., 2013; Abuduaini et al., 2024). Furthermore, the observed associations between genetic variants and fluid intelligence (RPM) highlight their value as an early marker of gene–cognition interactions. Although less commonly applied in MCI or AD diagnostics (Ruchinskas, 2019; Boccardi et al., 2022), intelligence measures capture interindividual variability in aging and are strongly heritable, with broad and largely domain-general genetic influences, since most genetic effects on cognition are considered general rather than domain-specific (Plomin and Deary, 2015; Plomin and Von Stumm, 2018). Finally, our findings support the decision to analyze males and females separately. Sex-specific pathways in cognitive aging–linked to inflammatory, metabolic and microglial differences–may contribute to increased vulnerability of women to neurodegeneration (Hanamsagar and Bilbo, 2016; Holland et al., 2013; Arnold et al., 2020) and modify the risk of AD-related cognitive decline (Buckley et al., 2018). Some of our findings contrast with previous reports suggesting a stronger effect of *APOE* ε 4 in women than in men on cognitive outcomes in aging and AD, a discrepancy that may reflect differences in sample characteristics, race, diagnostic criteria, study design, and the specific cognitive domains assessed (Altmann et al., 2014; Neu et al., 2017; Barnes et al., 2013; Sundermann et al., 2018; Eid et al., 2019; Fan et al., 2019).

Several limitations should be considered when interpreting the present findings, particularly given the exploratory nature of this study and its focus on genetic influences on cognitive functioning. First, the relatively small sample size, particularly after stratification by sex and cognitive cluster, limited the statistical power to detect subtle effects and restricted the generalizability of the findings. However, an *a priori* power analysis indicated that the resulting cluster sizes were close to recommended thresholds, exceeding the minimum commonly cited for subgroup analyses. Thus, while the sample was not optimal for detecting smaller effects, it was adequate for exploratory regression analyses at the cluster level. Replication in larger datasets is essential. Furthermore, the scope of neuropsychological evaluation was limited, focusing primarily on selected cognitive domains such as memory, and biomarker data were not included. Second, the phenotype-to-genotype approach may have introduced a selection bias. Although this strategy helps identify specific cognitive effects of known variants, it may overlook subtler phenotypic variation and lead to biased estimates of variant penetrance and pathogenicity (Wilczewski et al., 2023). Third, this study used only the *Agglomerative Clustering* algorithm for unsupervised learning. Alternative approaches, including Density-Based Spatial Clustering of Applications with Noise (DBSCAN) and K-means, were tested but produced suboptimal results (see Supplementary Material 1.6). DBSCAN was highly sensitive to parameter settings and did not separate clusters with heterogeneous densities, often collapsing the data into a single dominant group (Ester et al., 1996). The K-means, which assume spherical clusters and equal variance, yielded unstable solutions and small clusters below the power analysis threshold, limiting the interpretability (Chong, 2021; Dalmaijer et al., 2022; Brydges, 2019). Future research should include a broader spectrum of cognitive and non-cognitive variables, such as personality traits (e.g., neuroticism), to better capture gene–behavior interactions (Hindley et al., 2023). Although no association has been observed between the *APOE* ε *4* allele and affective symptoms in Polish patients with AD (Gabryelewicz et al., 2002), incorporating such measures may improve understanding of cognitive–emotional profiles in preclinical stages. Moreover, explainable machine learning methods (e.g., SHapley Additive exPlanations values) could help unravel the relative impact of *APOE* ε *4*, *PICALM GG*, and their interplay with age, education and other modifiable risk factors (Lundberg and Lee, 2017; Fan et al., 2019).

## 5 Conclusion

This study suggests that the effects of the *APOE* ε3ε *4* allele and the *PICALM GG* variant on cognitive functioning may vary depending on sex. In lower-performing females, the *APOE* ε3ε *4* allele was associated with less precise recognition and more perseverative errors; in males, with reduced fluid intelligence. Additionally, in males, *APOE* × *PICALM* interactions affected delayed recall and recognition, indicating combined genetic effects on memory retrieval. A higher number of significant interactions for the *APOE* variant was observed in both the female and male groups. The *PICALM GG* genotype was less frequently involved in significant interactions independently, but its relevance increased significantly when interacting with *APOE*, highlighting the utility of such interaction-based analyses in future AD research. In particular, three-way interactions (gene × age × education) were observed more frequently in the male group. The clustering approach revealed subtle cognitive differences that remain within normative ranges. Longitudinal studies are needed to determine whether these patterns reflect early vulnerability or normal variability. Genetic effects should not be viewed as directly “encoding” cognition; instead, they influence cognitive outcomes through complex molecular, cellular, and environmental interactions.

## Data Availability

Publicly available datasets were analyzed in this study. This data can be found here: https://openneuro.org/datasets/ds004796/versions/1.0.4. The code is available from the authors upon request.

## References

[B1] Abuduaini Y. Chen W. Kong X.-Z. (2024). Handedness in alzheimer’s disease: a systematic review. Brain Res. 1840, 149131. 10.1016/j.brainres.2024.149131 39053686

[B2] Altmann A. Tian L. Henderson V. W. Greicius M. D. Investigators A. D. N. I. (2014). Sex modifies the apoe-related risk of developing alzheimer disease. Ann. Neurol. 75, 563–573. 10.1002/ana.24135 24623176 PMC4117990

[B3] Ando K. Nagaraj S. Küçükali F. De Fisenne M.-A. Kosa A.-C. Doeraene E. (2022). Picalm and Alzheimer’s disease: an update and perspectives. Cells 11, 3994. 10.3390/cells11243994 36552756 PMC9776874

[B4] Arnold M. Nho K. Kueider-Paisley A. Massaro T. Huynh K. Brauner B. (2020). Sex and apoe *ɛ*4 genotype modify the alzheimer’s disease serum metabolome. Nat. Commun. 11, 1148. 10.1038/s41467-020-14959-w 32123170 PMC7052223

[B5] Ayorech Z. Krapohl E. Plomin R. Von Stumm S. (2017). Genetic influence on intergenerational educational attainment. Psychol. Sci. 28, 1302–1310. 10.1177/0956797617707270 28715641 PMC5595239

[B6] Barnes L. Arvanitakis Z. Yu L. Kelly J. De Jager P. Bennett D. (2013). Apolipoprotein e and change in episodic memory in blacks and whites. Neuroepidemiology 40, 211–219. 10.1159/000342778 23364031 PMC3645297

[B7] Barral S. Bird T. Goate A. Farlow M. Diaz-Arrastia R. Bennett D. (2012). Genotype patterns at picalm, cr1, bin1, clu, and apoe genes are associated with episodic memory. Neurology 78, 1464–1471. 10.1212/WNL.0b013e3182553c48 22539578 PMC3345618

[B8] Bellenguez C. Küçükali F. Jansen I. E. Kleineidam L. Moreno-Grau S. Amin N. (2022). New insights into the genetic etiology of Alzheimer’s disease and related dementias. Nat. Genetics 54, 412–436. 10.1038/s41588-022-01024-z 35379992 PMC9005347

[B9] Benjamini Y. Hochberg Y. (1995). Controlling the false discovery rate: a practical and powerful approach to multiple testing. J. R. Statistical Soc. Series B Methodol. 57, 289–300. 10.1111/j.2517-6161.1995.tb02031.x

[B10] Boccardi M. Monsch A. U. Ferrari C. Altomare D. Berres M. Bos I. (2022). Harmonizing neuropsychological assessment for mild neurocognitive disorders in Europe. Alzheimer’s Dementia 18, 29–42. 10.1002/alz.12365 33984176 PMC9642857

[B11] Brydges C. R. (2019). Effect size guidelines, sample size calculations, and statistical power in gerontology. Innovation Aging 3, igz036. 10.1093/geroni/igz036 31528719 PMC6736231

[B12] Buckley R. F. Mormino E. C. Amariglio R. E. Properzi M. J. Rabin J. S. Lim Y. Y. (2018). Sex, amyloid, and apoe *ɛ*4 and risk of cognitive decline in preclinical alzheimer’s disease: findings from three well-characterized cohorts. Alzheimer’s Dementia 14, 1193–1203. 10.1016/j.jalz.2018.04.010 29803541 PMC6131023

[B13] Bunce D. Bielak A. A. Anstey K. J. Cherbuin N. Batterham P. J. Easteal S. (2014). Apoe genotype and cognitive change in young, middle-aged, and older adults living in the community. J. Gerontol. Ser. A Biomed. Sci. Med. Sci. 69, 379–386. 10.1093/gerona/glt103 23902936

[B14] Caselli R. J. Dueck A. C. Osborne D. Sabbagh M. N. Connor D. J. Ahern G. L. (2009). Longitudinal growth modeling of cognitive aging and the apoe e4 effect. N. Engl. J. Med. 361, 255–263. 10.1056/NEJMoa0809437 19605830 PMC2928998

[B15] Chang Y.-T. Huang C.-W. Huang S.-H. Hsu S.-W. Chang W.-N. Lee J.-J. (2019). Genetic interaction is associated with lower metabolic connectivity and memory impairment in clinically mild Alzheimer’s disease. Genes Brain Behav. 18, e12490. 10.1111/gbb.12490 29883038

[B16] Chang Y.-L. Zhuo Y.-Y. Luo D.-H. (2021). Education moderates the negative effect of apolipoprotein E ɛ4 on response inhibition in older adults. J. Alzheimer’s Dis. 82, 1147–1157. 10.3233/JAD-210183 34151797

[B17] Chong B. (2021). K-means clustering algorithm: a brief review. Acad. J. Comput. Inf. Sci. 4, 37–40.

[B18] Dalmaijer E. S. Nord C. L. Astle D. E. (2022). Statistical power for cluster analysis. BMC Bioinform. 23, 205. 10.1186/s12859-022-04675-1 35641905 PMC9158113

[B19] Delis D. C. Kaplan E. Kramer J. H. Ober B. A. (2008). California verbal learning test (cvlt) (ECPA United States).

[B20] Dzianok P. Kublik E. (2023a). Altered granulocyte count and erythrocyte measures in middle-aged, healthy carriers of apoe and picalm risk genes for Alzheimer’s disease. Acta Neurobiol. Exp. 83, 127–139. 10.55782/ane-2023-012 37493530

[B21] Dzianok P. Kublik E. (2023b). Alzheimer’s disease risk alleles of picalm gene may accelerate cognitive (memory and attention) performance in middle-aged healthy apoe-*ɛ*4 carriers. Alzheimer’s Dementia 19, e067545. 10.1002/alz.067545

[B22] Dzianok P. Kublik E. (2024). Pearl-neuro database: EEG, fMRI, health and lifestyle data of middle-aged people at risk of dementia. Sci. Data 11, (1) 276. 10.1038/s41597-024-03106-5 38453963 PMC10920678

[B23] Dzianok P. Wojciechowski J. Wolak T. Kublik E. (2024). Alzheimer’s disease-like features in resting state eeg/fmri of cognitively intact and healthy middle-aged apoe/picalm risk carriers. bioRxiv. 10.1101/2024.06.20.599857 PMC12231819 40095677

[B25] Edlin J. M. Leppanen M. L. Fain R. J. Hackländer R. P. Hanaver-Torrez S. D. Lyle K. B. (2015). On the use (and misuse?) of the edinburgh handedness inventory. Brain Cognition 94, 44–51. 10.1016/j.bandc.2015.01.003 25656540

[B26] Eid A. Mhatre I. Richardson J. R. (2019). Gene-environment interactions in Alzheimer’s disease: a potential path to precision medicine. Pharmacol. Therapeutics 199, 173–187. 10.1016/j.pharmthera.2019.03.005 30877021 PMC6827882

[B27] Elwood R. W. (1995). The California verbal learning test: psychometric characteristics and clinical application. Neuropsychol. Rev. 5, 173–201. 10.1007/BF02214761 8653108

[B28] Engelman C. D. Koscik R. L. Jonaitis E. M. Okonkwo O. C. Hermann B. P. La Rue A. (2013). Interaction between two cholesterol metabolism genes influences memory: findings from the Wisconsin registry for Alzheimer’s prevention. J. Alzheimer’s Dis. 36, 749–757. 10.3233/JAD-130482 23669301 PMC3759032

[B29] Ester M. Kriegel H.-P. Sander J. Xu X. (1996). A density-based algorithm for discovering clusters in large spatial databases with noise. Kdd 96, 226–231.

[B30] Fan J. Tao W. Li X. Li H. Zhang J. Wei D. (2019). The contribution of genetic factors to cognitive impairment and dementia: apolipoprotein e gene, gene interactions, and polygenic risk. Int. J. Mol. Sci. 20, 1177. 10.3390/ijms20051177 30866553 PMC6429136

[B31] Furney S. Simmons A. Breen G. Pedroso I. Lunnon K. Proitsi P. (2011). Genome-wide association with mri atrophy measures as a quantitative trait locus for alzheimer’s disease. Mol. Psychiatry 16, 1130–1138. 10.1038/mp.2010.123 21116278 PMC5980656

[B32] Gabryelewicz T. Religa D. Styczynska M. Peplonska B. Pfeffer A. Wasiak B. (2002). Behavioural pathology in alzheimer’s disease with special reference to apolipoprotein e genotype. Dementia Geriatric Cognitive Disord. 14, 208–212. 10.1159/000066020 12411763

[B33] Greenacre M. Groenen P. J. Hastie T. d’Enza A. I. Markos A. Tuzhilina E. (2022). Principal component analysis. Nat. Rev. Methods Prim. 2, 100. 10.1038/s43586-022-00184-w

[B24] Haley G. E. Berteau-Pavy F. Park B. Raber J. (2010). Effects of *ɛ*4 on object recognition in the non-demented elderly. Curr. Aging Sci. 3, (2) 127–137. 10.2174/1874609811003020127 20044903 PMC4849126

[B34] Hanamsagar R. Bilbo S. D. (2016). Sex differences in neurodevelopmental and neurodegenerative disorders: focus on microglial function and neuroinflammation during development. J. Steroid Biochem. Mol. Biol. 160, 127–133. 10.1016/j.jsbmb.2015.09.039 26435451 PMC4829467

[B35] Hindley G. Shadrin A. A. van der Meer D. Parker N. Cheng W. O’Connell K. S. (2023). Multivariate genetic analysis of personality and cognitive traits reveals abundant pleiotropy. Nat. Human Behav. 7, 1584–1600. 10.1038/s41562-023-01630-9 37365406 PMC10824266

[B36] Hirono N. Hashimoto M. Yasuda M. Kazui H. Mori E. (2003). Accelerated memory decline in alzheimer’s disease with apolipoprotein *ɛ*4 allele. J. Neuropsychiatry Clin. Neurosci. 15, 354–358. 10.1176/jnp.15.3.354 12928512

[B37] Holland D. Desikan R. S. Dale A. M. McEvoy L. K. , and Alzheimer's Disease Neuro, and imaging Initiative (2013). Higher rates of decline for women and apolipoprotein e *ɛ*4 carriers. Am. J. Neuroradiol. 34, 2287–2293. 10.3174/ajnr.A3601 23828104 PMC3894062

[B38] Hoogmartens J. Cacace R. Van Broeckhoven C. (2021). Insight into the genetic etiology of alzheimer’s disease: a comprehensive review of the role of rare variants. Alzheimer’s Dementia Diagnosis, Assess. Dis. Monit. 13, e12155. 10.1002/dad2.12155 33665345 PMC7896636

[B39] Hsu Y.-C. Lin M.-C. Su M.-H. Cheng C.-F. Pan Y.-J. Fan C. C. (2025). Education as a modifier of genetic influence on cognitive ability in older adults. Behav. Genet. 55, 384–394. 10.1007/s10519-025-10229-x 40839240

[B40] Huang Y. Mahley R. W. (2014). Apolipoprotein e: structure and function in lipid metabolism, neurobiology, and alzheimer’s diseases. Neurobiol. Dis. 72, 3–12. 10.1016/j.nbd.2014.08.025 25173806 PMC4253862

[B41] Huang Y. A. Zhou B. Nabet A. M. Wernig M. Südhof T. C. (2019). Differential signaling mediated by apoe2, apoe3, and apoe4 in human neurons parallels Alzheimer’s disease risk. J. Neurosci. 39, 7408–7427. 10.1523/JNEUROSCI.2994-18.2019 31331998 PMC6759032

[B42] Hubacek J. A. Piper B. J. Pikhart H. Peasey A. Kubinova R. Bobak M. (2013). Lack of an association between left-handedness and apoe polymorphism in a large sample of adults: results of the Czech hapiee study. Laterality Asymmetries Body Brain Cognition 18, 513–519. 10.1080/1357650X.2012.715164 23113606 PMC3996547

[B43] Izaks G. J. Gansevoort R. T. van der Knaap A. M. Navis G. Dullaart R. P. Slaets J. P. (2011). The association of apoe genotype with cognitive function in persons aged 35 years or older. PLoS One 6, e27415. 10.1371/journal.pone.0027415 22110642 PMC3215744

[B44] Jack Jr C. R. Wiste H. J. Lesnick T. G. Weigand S. D. Knopman D. S. Vemuri P. (2013). Brain *β*-amyloid load approaches a Plateau. Neurology 80, 890–896. 10.1212/WNL.0b013e3182840bbe 23446680 PMC3653215

[B45] Jansen I. E. Savage J. E. Watanabe K. Bryois J. Williams D. M. Steinberg S. (2019). Genome-wide meta-analysis identifies new loci and functional pathways influencing alzheimer’s disease risk. Nat. Genetics 51, 404–413. 10.1038/s41588-018-0311-9 30617256 PMC6836675

[B46] Jaworowska A. Szustrowa T. (2010). Test matryc ravena w wersji standard forma równoległa. Warszawa: Pracownia Testów Psychologicznych PTP.

[B47] Jochemsen H. M. Muller M. van der Graaf Y. Geerlings M. I. (2012). Apoe *ɛ*4 differentially influences change in memory performance depending on age. The smart-mr study. Neurobiol. Aging 33, 832–e15. 10.1016/j.neurobiolaging.2011.07.016 21908077

[B48] Jun G. Naj A. C. Beecham G. W. Wang L. S. Buros J. Gallins P. J. (2011). Meta-analysis confirms cr1, clu, and picalm as alzheimer disease risk loci and reveals interactions with apoe genotypes. Arch. Neurol. 67, (12) 1473–1484. 10.1001/archneurol.2010.201 20697030 PMC3048805

[B49] Lakens D. (2022). Sample size justification. Collabra Psychol. 8, 33267. 10.1525/collabra.33267

[B50] Lancaster C. Tabet N. Rusted J. (2016). The apoe paradox: do attentional control differences in mid-adulthood reflect risk of late-life cognitive decline. Neurobiol. Aging 48, 114–121. 10.1016/j.neurobiolaging.2016.08.015 27661410

[B51] Le Carret N. Lafont S. Letenneur L. Dartigues J.-F. Mayo W. Fabrigoule C. (2003). The effect of education on cognitive performances and its implication for the constitution of the cognitive reserve. Dev. Neuropsychol. 23, 317–337. 10.1207/S15326942DN2303_1 12740188

[B52] Li T. Rezaeipanah A. El Din E. M. T. (2022). An ensemble agglomerative hierarchical clustering algorithm based on clusters clustering technique and the novel similarity measurement. J. King Saud University-Computer Inf. Sci. 34, 3828–3842. 10.1016/j.jksuci.2022.04.010

[B53] Liu C.-C. Kanekiyo T. Xu H. Bu G. (2013). Apolipoprotein e and alzheimer disease: risk, mechanisms and therapy. Nat. Rev. Neurol. 9, 106–118. 10.1038/nrneurol.2012.263 23296339 PMC3726719

[B54] Liu Y.-B. Wang X.-J. Tan L. Tan C.-C. Xu W. Initiative A. D. N. (2023). Picalm variation moderates the relationships of apoe 4 with alzheimer’s disease cerebrospinal biomarkers and memory function among non-demented population. J. Alzheimer’s Dis. 96, 1651–1661. 10.3233/JAD-230516 38007652

[B55] Livingston G. Huntley J. Liu K. Y. Costafreda S. G. Selbæk G. Alladi S. (2024). Dementia prevention, intervention, and care: 2024 report of the lancet standing commission. Lancet 404, 572–628. 10.1016/S0140-6736(24)01296-0 39096926

[B56] Łojek E. Stańczak J. Delis D. C. Kramer J. H. Ober B. A. (2010). Podręcznik do kalifornijskiego testu uczenia się językowego CVLT Deana C. Delisa, Joela H. Kramera. Ed. Kaplan I Beth A. Ober Polska Normalizacja Prac. Testów Psychol. Pol. Tow. Psychol.

[B57] Luciano M. Gow A. J. Harris S. E. Hayward C. Allerhand M. Starr J. M. (2009). Cognitive ability at age 11 and 70 years, information processing speed, and apoe variation: the lothian birth cohort 1936 study. Psychol. Aging 24, 129–138. 10.1037/a0014780 19290744

[B58] Lundberg S. M. Lee S.-I. (2017). A unified approach to interpreting model predictions. Adv. Neural Information Processing Systems. 10.48550/arXiv.1705.07874

[B59] Mengel-From J. Christensen K. McGue M. Christiansen L. (2011). Genetic variations in the clu and picalm genes are associated with cognitive function in the oldest old. Neurobiol. Aging 32, 554–e7. 10.1016/j.neurobiolaging.2010.07.016 20739100 PMC3042035

[B60] Miozzo M. Fischer-Baum S. Caccappolo-van Vliet E. (2013). Perseverations in Alzheimer’s disease: memory slips? Cortex 49, 2028–2039. 10.1016/j.cortex.2012.10.016 23375443

[B61] Morgen K. Ramirez A. Frölich L. Tost H. Plichta M. M. Kölsch H. (2014). Genetic interaction of picalm and apoe is associated with brain atrophy and cognitive impairment in Alzheimer’s disease. Alzheimer’s Dementia 10, S269–S276. 10.1016/j.jalz.2013.11.001 24613704

[B62] Neu S. C. Pa J. Kukull W. Beekly D. Kuzma A. Gangadharan P. (2017). Apolipoprotein e genotype and sex risk factors for alzheimer disease: a meta-analysis. JAMA Neurol. 74, 1178–1189. 10.1001/jamaneurol.2017.2188 28846757 PMC5759346

[B63] Pakhomov S. V. Eberly L. E. Knopman D. S. (2018). Recurrent perseverations on semantic verbal fluency tasks as an early marker of cognitive impairment. J. Clin. Experi. Neuropsychol. 40, 832–840. 10.1080/13803395.2018.1438372 29502483 PMC6338072

[B64] Panico F. Sagliano L. Magliacano A. Santangelo G. Trojano L. (2023). The relationship between cognitive reserve and cognition in healthy adults: a systematic review. Curr. Psychol. 42, 24751–24763. 10.1007/s12144-022-03523-y

[B65] Pettigrew C. Soldan A. Li S. Lu Y. Wang M.-C. Selnes O. A. (2013). Relationship of cognitive reserve and apoe status to the emergence of clinical symptoms in preclinical alzheimer’s disease. Cogn. Neurosci. 4, 136–142. 10.1080/17588928.2013.831820 24168200 PMC3836845

[B66] Pettigrew C. Nazarovs J. Soldan A. Singh V. Wang J. Hohman T. (2023). Alzheimer’s disease genetic risk and cognitive reserve in relationship to long-term cognitive trajectories among cognitively normal individuals. Alzheimer’s Res. Ther. 15, 66. 10.1186/s13195-023-01206-9 36978190 PMC10045505

[B67] Pinto C. Tandel K. Y. (2016). Cognitive reserve: concept, determinants, and promotion. J. Geriatric Mental Health 3, 44–51. 10.4103/2348-9995.181916

[B68] Plomin R. Deary I. J. (2015). Genetics and intelligence differences: five special findings. Mol. Psychiatry 20, 98–108. 10.1038/mp.2014.105 25224258 PMC4270739

[B69] Plomin R. Von Stumm S. (2018). The new genetics of intelligence. Nat. Rev. Genet. 19, 148–159. 10.1038/nrg.2017.104 29335645 PMC5985927

[B70] Ponomareva N. Andreeva T. Protasova M. Konovalov R. Krotenkova M. Malina D. (2020). Genetic association between alzheimer’s disease risk variant of the picalm gene and eeg functional connectivity in non-demented adults. Front. Neurosci. 14, 324. 10.3389/fnins.2020.00324 32372909 PMC7177435

[B71] Raulin A.-C. Doss S. V. Trottier Z. A. Ikezu T. C. Bu G. Liu C.-C. (2022). Apoe in alzheimer’s disease: pathophysiology and therapeutic strategies. Mol. Neurodegeneration 17, 72. 10.1186/s13024-022-00574-4 36348357 PMC9644639

[B72] Raven J. (2008). The raven progressive matrices tests: their theoretical basis and measurement model. Uses Abuses Intell. Stud. Advancing Spearman Raven’s Quest Non-arbitrary Metrics, 17–68.

[B73] Raven J. (2003). Raven progressive matrices. Handbook of nonverbal assessment. Boston, MA: Springer.

[B74] Reinvang I. Winjevoll I. L. Rootwelt H. Espeseth T. (2010). Working memory deficits in healthy apoe epsilon 4 carriers. Neuropsychologia 48, 566–573. 10.1016/j.neuropsychologia.2009.10.018 19879282

[B75] Religa D. Styczynska M. Peplonska B. Gabryelewicz T. Pfeffer A. Chodakowska M. (2003). Homocysteine, apolipoproteine e and methylenetetrahydrofolate reductase in alzheimer’s disease and mild cognitive impairment. Dementia Geriatric Cognitive Disorders 16, 64–70. 10.1159/000070677 12784029

[B76] Ruchinskas R. (2019). Wechsler adult intelligence scale-digit span performance in subjective cognitive complaints, amnestic mild cognitive impairment, and probable dementia of the Alzheimer type. Clin. Neuropsychologist 33, 1436–1444. 10.1080/13854046.2019.1585574 30931811

[B77] Rusted J. Evans S. King S. Dowell N. Tabet N. Tofts P. (2013). Apoe e4 polymorphism in young adults is associated with improved attention and indexed by distinct neural signatures. Neuroimage 65, 364–373. 10.1016/j.neuroimage.2012.10.010 23063453

[B78] Sampedro F. Vilaplana E. De Leon M. J. Alcolea D. Pegueroles J. Montal V. (2015). Apoe-by-sex interactions on brain structure and metabolism in healthy elderly controls. Oncotarget 6, 26663–26674. 10.18632/oncotarget.5185 26397226 PMC4694943

[B79] Sasirekha K. Baby P. (2013). Agglomerative hierarchical clustering algorithm a review. Int. J. Sci. Res. Publ. 3 (3), 1–3.

[B80] Scheltens N. M. Galindo-Garre F. Pijnenburg Y. A. van der Vlies A. E. Smits L. L. Koene T. (2016). The identification of cognitive subtypes in Alzheimer’s disease dementia using latent class analysis. J. Neurol. Neurosurg. Psychiatry 87, 235–243. 10.1136/jnnp-2014-309582 25783437

[B81] Schultz M. R. Lyons M. J. Franz C. E. Grant M. D. Boake C. Jacobson K. C. (2008). Apolipoprotein e genotype and memory in the sixth decade of life. Neurology 70, 1771–1777. 10.1212/01.wnl.0000286941.74372.cc 18235080 PMC3107734

[B82] Singhal P. Verma S. S. Ritchie M. D. (2023). Gene interactions in human disease studies–evidence is mounting. Annu. Rev. Biomed. Data Sci. 6, 377–395. 10.1146/annurev-biodatasci-102022-120818 37196359

[B83] Styczynska M. Religa D. Pfeffer A. Luczywek E. Wasiak B. Styczynski G. (2003). Simultaneous analysis of five genetic risk factors in polish patients with Alzheimer’s disease. Neurosci. Letters 344, 99–102. 10.1016/s0304-3940(03)00438-5 12782337

[B84] Sun D.-M. Chen H.-F. Zuo Q.-L. Su F. Bai F. Liu C.-F. (2017). Effect of picalm rs3851179 polymorphism on the default mode network function in mild cognitive impairment. Behav. Brain Res. 331, 225–232. 10.1016/j.bbr.2017.05.043 28549650

[B85] Sundermann E. E. Tran M. Maki P. M. Bondi M. W. Initiative A. D. N. (2018). Sex differences in the association between apolipoprotein e *ɛ*4 allele and alzheimer’s disease markers. Alzheimer’s Dementia Diagnosis Assess. Dis. Monit. 10, 438–447. 10.1016/j.dadm.2018.06.004 30182053 PMC6120724

[B86] Sweet R. A. Seltman H. Emanuel J. E. Lopez O. L. Becker J. T. Bis J. C. (2012). Effect of Alzheimer’s disease risk genes on trajectories of cognitive function in the cardiovascular health study. Am. J. Psychiatry 169, 954–962. 10.1176/appi.ajp.2012.11121815 22952074 PMC3610571

[B87] Thomas K. R. Eppig J. Edmonds E. C. Jacobs D. M. Libon D. J. Au R. (2018). Word-list intrusion errors predict progression to mild cognitive impairment. Neuropsychology 32, 235–245. 10.1037/neu0000413 29528684 PMC5851458

[B88] Torres V. L. Rosselli M. Loewenstein D. A. Curiel R. E. Vélez Uribe I. Lang M. (2019). Types of errors on a semantic interference task in mild cognitive impairment and dementia. Neuropsychology 33, 670–684. 10.1037/neu0000542 31070384 PMC6731098

[B89] Tuminello E. R. Han S. D. (2011). The apolipoprotein e antagonistic pleiotropy hypothesis: review and recommendations. Int. J. Alzheimer’s Disease 2011, 726197. 10.4061/2011/726197 21423560 PMC3056453

[B90] Vonk J. M. Arce Rentería M. Medina V. M. Pericak-Vance M. A. Byrd G. S. Haines J. (2019). Education moderates the relation between apoe 4 and memory in nondemented Non-hispanic black older adults. J. Alzheimer’s Dis. 72, 495–506. 10.3233/JAD-190415 31594222 PMC8876947

[B91] Walsemann K. M. Jackson H. M. Boardman J. D. Herd P. (2025). Apoe genotype and cognitive decline: educational context as a moderator of genetic risk. J. Gerontol. Ser. B Psychol. Sci. Soc. Sci. 80, gbaf110. 10.1093/geronb/gbaf110 40580546 PMC12296396

[B92] Wang Y. Liu S. Spiteri A. G. Huynh A. L. H. Chu C. Masters C. L. (2024). Understanding machine learning applications in dementia research and clinical practice: a review for biomedical scientists and clinicians. Alzheimer’s Res. Therapy 16, 175. 10.1186/s13195-024-01540-6 39085973 PMC11293066

[B93] Wilczewski C. M. Obasohan J. Paschall J. E. Zhang S. Singh S. Maxwell G. L. (2023). Genotype first: clinical genomics research through a reverse phenotyping approach. Am. J. Hum. Genet. 110, 3–12. 10.1016/j.ajhg.2022.12.004 36608682 PMC9892776

[B94] Wu Z. Yang Y. Song Z. Ma M. Feng M. Liu Y. (2022). Picalm rs3851179 variants modulate left postcentral cortex thickness, csf amyloid *β*42, and phosphorylated tau in the elderly. Brain Sci. 12, 1681. 10.3390/brainsci12121681 36552141 PMC9776362

[B95] Wu Z. Chen J. Liu Y. Yang Y. Feng M. Dai H. (2024). The effects of picalm rs3851179 and age on brain atrophy and cognition along the Alzheimer’s disease continuum. Mol. Neurobiol. 61, 6984–6996. 10.1007/s12035-024-03953-8 38363532

[B96] Xu W. Tan C.-C. Cao X.-P. Tan L. Initiative A. D. N. (2020). Association of Alzheimer’s disease risk variants on the picalm gene with picalm expression, core biomarkers, and feature neurodegeneration. Aging (Albany NY) 12, 21202–21219. 10.18632/aging.103814 33170153 PMC7695360

[B97] Zeng F.-F. Liu J. He H. Gao X.-P. Liao M.-Q. Yu X.-X. (2019). Association of picalm gene polymorphisms with Alzheimer’s disease: evidence from an updated meta-analysis. Curr. Alzheimer Res. 16, 1196–1205. 10.2174/1567205016666190805165607 31385771

