# Supplementary material for: Sex differences in APOE- and PICALM-related cognitive profiles in healthy middle-aged adults
Source: Front Aging. 2026 Jan 13;6:1694701. doi: 10.3389/fragi.2025.1694701 (PMC12835333; doi:10.3389/fragi.2025.1694701)
Supplement: Supplementary file 1 [file DataSheet1.pdf]

Supplementary Material

1 SUPPLEMENTARY TABLES AND FIGURES

1.1 Models of interactions; cognitive tests for better and worse clusters

Table S1. Female's Cognitive Test Results for Better and Worse Clusters: Model 3, APOE × PICALM (significant *p*-values highlighted)

Variable	Better Cluster				Worse Cluster			
	Coef.	95% CI	*p*	R^2	Coef.	95% CI	*p*	R^2
CVLT_1	-4.74	[-10.33, 0.85]	0.0949	0.081	0.34	[-10.37, 11.05]	0.9491	0.0003
CVLT_2	-2.01	[-4.04, 0.03]	0.0530	0.108	-0.75	[-3.45, 1.96]	0.5810	0.0099
CVLT_3	-0.37	[-1.54, 0.80]	0.5304	0.023	0.25	[-2.04, 2.54]	0.8262	0.0153
CVLT_4	-0.52	[-3.00, 1.95]	0.6735	0.004	2.08	[-0.72, 4.88]	0.1406	0.0819
CVLT_5	0.05	[-1.11, 1.21]	0.9347	0.016	0.98	[-1.93, 3.88]	0.4997	0.0359
CVLT_6	-0.13	[-1.07, 0.82]	0.7855	0.030	0.90	[-0.83, 2.64]	0.2970	0.0470
CVLT_7	-0.27	[-1.16, 0.62]	0.5442	0.011	1.44	[-1.22, 4.09]	0.2798	0.0522
CVLT_8	0.01	[-0.94, 0.95]	0.9900	0.017	0.72	[-1.39, 2.83]	0.4955	0.0278
CVLT_9	-1.17	[-6.56, 4.23]	0.6666	0.053	-0.76	[-8.51, 7.00]	0.8444	0.1868
CVLT_10	1.20	[0.22, 2.18]	0.0177	0.102	-0.06	[-3.04, 2.93]	0.9693	0.2323
CVLT_11	0.79	[0.23, 1.34]	0.0065	0.133	-0.70	[-2.14, 0.74]	0.3330	0.1945
CVLT_12	0.19	[-0.20, 0.59]	0.3356	0.031	0.54	[-0.82, 1.90]	0.4288	0.1488
CVLT_13	0.12	[-0.10, 0.34]	0.2759	0.076	-0.61	[-2.74, 1.51]	0.5612	0.0348
RPM	0.85	[-5.02, 6.72]	0.7720	0.008	4.58	[-2.88, 12.04]	0.2214	0.0419
EHI	-5.74	[-23.51, 12.04]	0.5207	0.032	6.71	[-33.05, 46.46]	0.7348	0.0560

Table S2. Male's Cognitive Test Results for Better and Worse Clusters: Model 3, APOE × PICALM (significant *p*-values highlighted)

Variable	Better Cluster				Worse Cluster			
	Coef.	95% CI	*p*	R^2	Coef.	95% CI	*p*	R^2
CVLT_1	-5.04	[-12.87, 2.79]	0.2000	0.062	-13.43	[-23.35, -3.51]	0.0091	0.160
CVLT_2	-0.77	[-3.62, 2.08]	0.5869	0.055	-2.15	[-4.42, 0.11]	0.0621	0.109
CVLT_3	-0.21	[-2.16, 1.74]	0.8286	0.004	-3.43	[-6.28, -0.58]	0.0193	0.160
CVLT_4	0.31	[-2.65, 3.28]	0.8306	0.004	-1.83	[-4.37, 0.72]	0.1563	0.070
CVLT_5	1.73	[-0.87, 4.33]	0.1845	0.051	-3.79	[-7.45, -0.13]	0.0428	0.102
CVLT_6	0.59	[-1.09, 2.27]	0.4813	0.018	-3.60	[-6.48, -0.73]	0.0152	0.127
CVLT_7	-0.38	[-1.94, 1.17]	0.6195	0.044	-3.22	[-6.85, 0.41]	0.0808	0.090
CVLT_8	0.03	[-1.71, 1.76]	0.9757	0.029	-3.99	[-7.11, -0.87]	0.0132	0.130
CVLT_9	0.05	[-2.85, 2.95]	0.9722	0.043	-2.18	[-8.11, 3.74]	0.4624	0.023
CVLT_10	1.57	[0.14, 3.01]	0.0324	0.139	-0.18	[-2.91, 2.54]	0.8922	0.017
CVLT_11	-0.66	[-2.21, 0.89]	0.3929	0.077	3.51	[0.11, 6.91]	0.0434	0.133
CVLT_12	0.55	[-0.17, 1.26]	0.1291	0.074	-1.68	[-3.86, 0.50]	0.1276	0.105
CVLT_13	-0.33	[-0.96, 0.30]	0.3010	0.048	2.93	[1.00, 4.86]	0.0038	0.235
RPM	0.38	[-4.21, 4.96]	0.8678	0.008	0.46	[-5.13, 6.05]	0.8703	0.101
EHI	13.31	[-11.13, 37.75]	0.2762	0.093	-5.38	[-38.69, 27.92]	0.7464	0.086

Table S3. Female's Cognitive Test Results for Better and Worse Clusters, Model 3a, APOE × PICALM × age (significant *p*-values highlighted).

Variable	Better Cluster				Worse Cluster			
	Coef.	95% CI	*p*	R^2	Coef.	95% CI	*p*	R^2
CVLT_1	-0.24	[-2.27, 1.78]	0.8102	0.124	2.32	[-2.24, 6.89]	0.3085	0.118
CVLT_2	-0.00	[-0.73, 0.72]	0.9909	0.182	0.12	[-1.07, 1.31]	0.8393	0.077
CVLT_3	0.02	[-0.41, 0.44]	0.9421	0.078	0.70	[-0.23, 1.63]	0.1371	0.205
CVLT_4	0.61	[-0.27, 1.50]	0.1711	0.078	-0.08	[-1.27, 1.12]	0.8991	0.188
CVLT_5	-0.05	[-0.44, 0.33]	0.7873	0.207	0.94	[-0.26, 2.13]	0.1197	0.213
CVLT_6	-0.08	[-0.42, 0.25]	0.6192	0.104	0.30	[-0.48, 1.07]	0.4420	0.082
CVLT_7	0.01	[-0.30, 0.33]	0.9417	0.090	0.52	[-0.60, 1.64]	0.3546	0.175
CVLT_8	0.04	[-0.30, 0.38]	0.8255	0.061	1.08	[0.22, 1.94]	0.01487	0.225
CVLT_9	-0.31	[-2.28, 1.66]	0.7527	0.084	2.82	[-0.54, 6.19]	0.0977	0.257
CVLT_10	-0.03	[-0.38, 0.32]	0.8501	0.173	-0.26	[-1.58, 1.06]	0.6871	0.272
CVLT_11	-0.01	[-0.22, 0.20]	0.9159	0.140	-0.14	[-0.72, 0.43]	0.6180	0.376
CVLT_12	0.03	[-0.12, 0.17]	0.6976	0.057	-0.88	[-1.96, 0.20]	0.1072	0.331
CVLT_13	0.04	[-0.04, 0.12]	0.2690	0.110	0.54	[-1.15, 2.23]	0.5234	0.234
RPM	0.40	[-1.38, 2.17]	0.6570	0.340	-1.31	[-4.52, 1.90]	0.4140	0.140
EHI	-2.43	[-8.83, 3.97]	0.4497	0.089	9.14	[-7.39, 25.66]	0.2693	0.209

Table S4. Male's Cognitive Test Results for Better and Worse Clusters, Model 3a, APOE × PICALM × age (significant *p*-values highlighted)

Variable	Better Cluster				Worse Cluster			
	Coef.	95% CI	*p*	R^2	Coef.	95% CI	*p*	R^2
CVLT_1	-0.59	[-3.10, 1.92]	0.6357	0.152	-0.76	[-4.16, 2.64]	0.6532	0.208
CVLT_2	-0.37	[-1.30, 0.56]	0.4228	0.121	-0.80	[-1.55, -0.06]	0.0355	0.219
CVLT_3	-0.07	[-0.71, 0.57]	0.8315	0.060	0.19	[-0.74, 1.11]	0.6881	0.282
CVLT_4	-0.24	[-1.14, 0.66]	0.5888	0.193	0.03	[-0.84, 0.89]	0.9537	0.138
CVLT_5	0.09	[-0.70, 0.88]	0.8181	0.233	0.25	[-0.97, 1.47]	0.6859	0.198
CVLT_6	0.01	[-0.54, 0.57]	0.9578	0.048	-0.35	[-1.27, 0.57]	0.4482	0.284
CVLT_7	0.03	[-0.49, 0.55]	0.9088	0.060	-0.69	[-1.88, 0.50]	0.2505	0.212
CVLT_8	0.05	[-0.53, 0.63]	0.8510	0.039	-0.99	[-1.91, -0.08]	0.0343	0.396
CVLT_9	-0.14	[-1.06, 0.78]	0.7519	0.149	0.10	[-1.95, 2.14]	0.9227	0.066
CVLT_10	0.35	[-0.10, 0.81]	0.1238	0.230	0.63	[-0.28, 1.54]	0.1699	0.118
CVLT_11	-0.41	[-0.88, 0.05]	0.0801	0.260	1.67	[0.78, 2.55]	0.0005	0.526
CVLT_12	0.07	[-0.16, 0.31]	0.5375	0.108	0.12	[-0.63, 0.87]	0.7475	0.147
CVLT_13	-0.19	[-0.35, -0.02]	0.0289	0.416	0.86	[0.35, 1.36]	0.0014	0.580
RPM	-0.82	[-2.29, 0.66]	0.2676	0.097	-0.72	[-2.58, 1.14]	0.4413	0.199
EHI	5.88	[-1.81, 13.56]	0.1287	0.211	-1.07	[-12.74, 10.59]	0.8535	0.099

Table S5. Female's Cognitive Test Results for Better and Worse Clusters, Model 3b, APOE × PICALM × education (significant *p*-values highlighted)

Variable	Better Cluster				Worse Cluster			
	Coef.	95% CI	*p*	R^2	Coef.	95% CI	*p*	R^2
CVLT_1	-1.43	[-3.22, 0.35]	0.1132	0.126	-0.20	[-3.95, 3.55]	0.9139	0.040
CVLT_2	-0.62	[-1.26, 0.03]	0.0595	0.147	-0.36	[-1.29, 0.58]	0.4449	0.133
CVLT_3	-0.13	[-0.50, 0.23]	0.4673	0.049	0.05	[-0.79, 0.90]	0.9030	0.023
CVLT_4	-0.02	[-0.76, 0.71]	0.9549	0.112	0.40	[-0.58, 1.37]	0.4146	0.156
CVLT_5	0.08	[-0.28, 0.44]	0.6479	0.083	0.24	[-0.80, 1.28]	0.6445	0.079
CVLT_6	0.01	[-0.29, 0.31]	0.9412	0.038	0.15	[-0.45, 0.75]	0.6123	0.118
CVLT_7	-0.03	[-0.31, 0.26]	0.8586	0.060	0.27	[-0.69, 1.23]	0.5717	0.082
CVLT_8	0.03	[-0.27, 0.33]	0.8478	0.058	0.18	[-0.59, 0.95]	0.6339	0.049
CVLT_9	-0.31	[-2.05, 1.44]	0.7252	0.096	0.39	[-1.79, 2.56]	0.7213	0.460
CVLT_10	0.36	[0.04, 0.68]	0.0302	0.116	-0.20	[-1.25, 0.85]	0.7023	0.301
CVLT_11	0.22	[0.03, 0.40]	0.02067	0.167	-0.23	[-0.75, 0.29]	0.3707	0.208
CVLT_12	0.09	[-0.04, 0.22]	0.1654	0.099	0.22	[-0.21, 0.65]	0.3089	0.352
CVLT_13	0.03	[-0.04, 0.10]	0.3725	0.081	0.19	[-0.49, 0.87]	0.5668	0.254
RPM	0.37	[-1.58, 2.32]	0.7026	0.017	0.95	[-1.25, 3.15]	0.3860	0.384
EHI	-1.50	[-7.16, 4.16]	0.5968	0.037	-1.42	[-15.50, 12.66]	0.8389	0.099

Table S6. Male's Cognitive Test Results for Better and Worse Clusters, Model 3b, APOE × PICALM × education (significant *p*-values highlighted)

Variable	Better Cluster				Worse Cluster			
	Coef.	95% CI	*p*	R^2	Coef.	95% CI	*p*	R^2
CVLT_1	-1.13	[-4.03, 1.76]	0.4295	0.080	9.52	[-3.19, 22.22]	0.1373	0.237
CVLT_2	-0.12	[-1.13, 0.90]	0.8112	0.143	-0.02	[-3.08, 3.03]	0.9873	0.204
CVLT_3	0.19	[-0.54, 0.91]	0.5994	0.070	1.16	[-2.81, 5.14]	0.5555	0.161
CVLT_4	0.40	[-0.62, 1.42]	0.4320	0.185	0.94	[-2.15, 4.04]	0.5399	0.236
CVLT_5	0.63	[-0.07, 1.33]	0.0767	0.376	5.58	[0.85, 10.31]	0.0220	0.249
CVLT_6	0.21	[-0.36, 0.78]	0.4557	0.195	3.90	[0.34, 7.45]	0.0325	0.250
CVLT_7	0.06	[-0.46, 0.57]	0.8171	0.255	5.64	[1.39, 9.89]	0.0107	0.275
CVLT_8	0.16	[-0.46, 0.79]	0.5996	0.191	4.33	[0.57, 8.09]	0.0253	0.325
CVLT_9	0.27	[-0.74, 1.27]	0.5917	0.197	6.57	[-0.05, 13.19]	0.0516	0.306
CVLT_10	0.39	[-0.14, 0.92]	0.1452	0.157	2.48	[-1.56, 6.52]	0.2207	0.068
CVLT_11	0.06	[-0.31, 0.43]	0.7274	0.037	1.15	[-4.01, 6.31]	0.6539	0.166
CVLT_12	0.17	[-0.09, 0.43]	0.1959	0.090	-0.24	[-3.04, 2.57]	0.8648	0.135
CVLT_13	-0.04	[-0.26, 0.18]	0.7206	0.044	-1.28	[-4.13, 1.58]	0.3702	0.304
RPM	0.28	[-1.37, 1.92]	0.7327	0.130	3.79	[-2.90, 10.47]	0.2580	0.227
EHI	2.59	[-3.59, 8.76]	0.3978	0.590	-10.86	[-57.78, 36.07]	0.6415	0.113

Table S7. Female's Cognitive Test Results for Better and Worse Clusters, Model 3c, APOE × PICALM × age × education (significant *p*-values highlighted)

Variable	Better Cluster				Worse Cluster			
	Coef.	95% CI	*p*	R^2	Coef.	95% CI	*p*	R^2
CVLT_1	-0.14	[-0.80, 0.53]	0.6811	0.176	1.37	[-0.30, 3.05]	0.1041	0.291
CVLT_2	-0.01	[-0.24, 0.22]	0.9095	0.244	0.24	[-0.21, 0.69]	0.2904	0.248
CVLT_3	-0.04	[-0.17, 0.10]	0.5815	0.097	0.27	[-0.10, 0.63]	0.1508	0.311
CVLT_4	0.18	[-0.09, 0.45]	0.1929	0.188	0.03	[-0.42, 0.47]	0.8966	0.352
CVLT_5	-0.03	[-0.16, 0.09]	0.5869	0.269	0.54	[0.14, 0.94]	0.0095	0.503
CVLT_6	-0.02	[-0.13, 0.08]	0.6639	0.173	0.29	[0.02, 0.55]	0.0356	0.367
CVLT_7	0.01	[-0.10, 0.11]	0.9096	0.139	0.43	[0.04, 0.82]	0.0325	0.442
CVLT_8	0.02	[-0.09, 0.13]	0.7520	0.103	0.52	[0.23, 0.80]	0.0010	0.514
CVLT_9	-0.08	[-0.74, 0.58]	0.8072	0.112	0.68	[-0.27, 1.63]	0.1515	0.622
CVLT_10	-0.03	[-0.14, 0.09]	0.6377	0.212	0.13	[-0.32, 0.58]	0.5599	0.517
CVLT_11	0.00	[-0.07, 0.07]	0.9405	0.208	-0.01	[-0.23, 0.21]	0.9004	0.476
CVLT_12	0.02	[-0.03, 0.06]	0.4832	0.257	-0.17	[-0.46, 0.11]	0.2284	0.656
CVLT_13	0.01	[-0.01, 0.04]	0.2816	0.119	0.03	[-0.55, 0.60]	0.9251	0.344
RPM	0.21	[-0.38, 0.81]	0.4740	0.372	0.53	[-0.41, 1.48]	0.2561	0.579
EHI	-0.80	[-2.84, 1.23]	0.4308	0.155	6.02	[0.29, 11.76]	0.0404	0.447

Table S8. Male's Cognitive Test Results for Better and Worse Clusters, Model 3c, APOE × PICALM × age × education (significant *p*-values highlighted)

Variable	Better Cluster				Worse Cluster			
	Coef.	95% CI	*p*	R^2	Coef.	95% CI	*p*	R^2
CVLT_1	-0.45	[-1.37, 0.46]	0.3178	0.218	0.17	[-0.95, 1.29]	0.7626	0.341
CVLT_2	-0.16	[-0.48, 0.17]	0.3343	0.246	-0.11	[-0.38, 0.16]	0.4045	0.299
CVLT_3	-0.09	[-0.32, 0.15]	0.4554	0.144	0.08	[-0.25, 0.40]	0.6417	0.368
CVLT_4	-0.12	[-0.45, 0.21]	0.4567	0.272	0.15	[-0.12, 0.42]	0.2735	0.338
CVLT_5	-0.07	[-0.29, 0.16]	0.5492	0.456	0.18	[-0.20, 0.56]	0.3417	0.453
CVLT_6	-0.06	[-0.24, 0.13]	0.5360	0.255	0.00	[-0.27, 0.27]	0.9812	0.517
CVLT_7	-0.07	[-0.24, 0.10]	0.4089	0.324	-0.08	[-0.41, 0.24]	0.6018	0.530
CVLT_8	-0.00	[-0.21, 0.20]	0.9632	0.259	-0.20	[-0.45, 0.04]	0.0974	0.685
CVLT_9	-0.10	[-0.41, 0.20]	0.4967	0.360	0.34	[-0.25, 0.93]	0.2447	0.396
CVLT_10	0.17	[0.01, 0.33]	0.0409	0.327	0.28	[-0.06, 0.63]	0.1031	0.247
CVLT_11	-0.04	[-0.15, 0.08]	0.5334	0.196	0.50	[0.15, 0.85]	0.0067	0.572
CVLT_12	0.02	[-0.07, 0.10]	0.6882	0.128	0.12	[-0.13, 0.38]	0.3282	0.210
CVLT_13	-0.05	[-0.10, 0.01]	0.0873	0.515	0.19	[-0.00, 0.37]	0.0501	0.673
RPM	-0.15	[-0.69, 0.40]	0.5844	0.191	0.18	[-0.38, 0.74]	0.5181	0.389
EHI	2.31	[0.53, 4.10]	0.0134	0.707	0.32	[-4.08, 4.73]	0.8814	0.130

Table S9. Female's Cognitive Test Results for Better and Worse Clusters, Model 1: APOE_e3e4 (significant *p*-values highlighted).

Variable	Better Cluster				Worse Cluster			
	Coef.	95% CI	*p*	R^2	Coef.	95% CI	*p*	R^2
CVLT_1	-0.60	[-3.41, 2.21]	0.6710	0.003	0.19	[-4.98, 5.36]	0.9418	0.000
CVLT_2	-0.58	[-1.61, 0.45]	0.2659	0.021	-0.03	[-1.34, 1.28]	0.9631	0.000
CVLT_3	0.27	[-0.30, 0.83]	0.3512	0.015	0.36	[-0.75, 1.46]	0.5176	0.010
CVLT_4	0.11	[-1.09, 1.31]	0.8535	0.001	0.21	[-1.20, 1.62]	0.7657	0.002
CVLT_5	-0.13	[-0.70, 0.43]	0.6376	0.004	0.70	[-0.71, 2.11]	0.3199	0.024
CVLT_6	-0.24	[-0.70, 0.21]	0.2909	0.019	-0.11	[-0.96, 0.75]	0.8034	0.002
CVLT_7	0.11	[-0.32, 0.54]	0.6066	0.005	-0.62	[-1.92, 0.68]	0.3428	0.022
CVLT_8	-0.13	[-0.59, 0.32]	0.5622	0.006	-0.41	[-1.44, 0.62]	0.4254	0.016
CVLT_9	-2.04	[-4.67, 0.58]	0.1244	0.040	5.59	[1.84, 9.34]	0.0045	0.181
CVLT_10	0.02	[-0.48, 0.52]	0.9297	0.000	-0.40	[-2.04, 1.24]	0.6272	0.006
CVLT_11	0.00	[-0.29, 0.29]	1.0000	0.000	0.20	[-0.57, 0.97]	0.5978	0.007
CVLT_12	-0.02	[-0.22, 0.17]	0.8202	0.001	-0.80	[-1.46, -0.14]	0.0193	0.129
CVLT_13	0.04	[-0.06, 0.15]	0.4149	0.012	0.15	[-0.88, 1.19]	0.7690	0.002
RPM	-0.76	[-3.60, 2.09]	0.5965	0.005	-0.39	[-4.06, 3.29]	0.8323	0.001
EHI	4.91	[-3.72, 13.55]	0.2596	0.022	5.50	[-14.16, 25.17]	0.5749	0.008

Table S10. Male's Cognitive Test Results for Better and Worse Clusters, Model 1: APOE_e3e4 (significant *p*-values highlighted).

Variable	Better Cluster				Worse Cluster			
	Coef.	95% CI	*p*	R^2	Coef.	95% CI	*p*	R^2
CVLT_1	1.31	[-2.47, 5.09]	0.4857	0.014	-0.63	[-5.80, 4.53]	0.8063	0.001
CVLT_2	0.68	[-0.68, 2.04]	0.3176	0.028	-0.79	[-1.92, 0.33]	0.1623	0.039
CVLT_3	0.13	[-0.79, 1.05]	0.7795	0.002	0.69	[-0.78, 2.16]	0.3484	0.018
CVLT_4	-0.19	[-1.59, 1.20]	0.7819	0.002	0.65	[-0.60, 1.90]	0.2996	0.022
CVLT_5	0.02	[-1.23, 1.28]	0.9698	0.000	-0.50	[-2.34, 1.35]	0.5913	0.006
CVLT_6	0.06	[-0.73, 0.86]	0.8716	0.001	0.31	[-1.15, 1.78]	0.6707	0.004
CVLT_7	0.30	[-0.45, 1.04]	0.4231	0.018	-0.88	[-2.68, 0.92]	0.3320	0.019
CVLT_8	0.39	[-0.43, 1.21]	0.3387	0.026	-0.48	[-2.07, 1.11]	0.5486	0.007
CVLT_9	-0.05	[-1.44, 1.34]	0.9418	0.000	0.96	[-1.89, 3.81]	0.5002	0.009
CVLT_10	-0.07	[-0.79, 0.66]	0.8521	0.001	-0.00	[-1.32, 1.31]	0.9941	0.000
CVLT_11	0.50	[-0.24, 1.24]	0.1775	0.050	0.93	[-0.79, 2.66]	0.2813	0.024
CVLT_12	-0.09	[-0.43, 0.26]	0.6124	0.007	0.59	[-0.50, 1.68]	0.2807	0.024
CVLT_13	-0.06	[-0.36, 0.25]	0.7047	0.004	0.87	[-0.15, 1.90]	0.0931	0.057
RPM	0.53	[-1.63, 2.69]	0.6249	0.007	-3.06	[-5.74, -0.38]	0.0258	0.097
EHI	-6.94	[-18.79, 4.92]	0.2432	0.038	15.14	[-0.92, 31.20]	0.0641	0.068

Table S11. Female's Cognitive Test Results for Better and Worse Clusters, Model 1a: APOE e3e4 (significant p-values highlighted)

Variable	Better Cluster				Worse Cluster			
	Coef.	95% CI	*p*	R^2	Coef.	95% CI	*p*	R^2
CVLT_1	-0.47	[-1.43, 0.49]	0.3345	0.025	0.89	[-1.04, 2.82]	0.3583	0.079
CVLT_2	-0.21	[-0.56, 0.14]	0.2279	0.048	0.16	[-0.34, 0.66]	0.5299	0.035
CVLT_3	0.07	[-0.12, 0.26]	0.4814	0.063	0.13	[-0.28, 0.55]	0.5182	0.082
CVLT_4	-0.19	[-0.60, 0.22]	0.3511	0.025	-0.01	[-0.55, 0.53]	0.9637	0.026
CVLT_5	-0.24	[-0.42, -0.05]	0.0127	0.116	0.14	[-0.38, 0.66]	0.5983	0.125
CVLT_6	-0.01	[-0.16, 0.15]	0.9470	0.079	-0.03	[-0.36, 0.30]	0.8463	0.025
CVLT_7	-0.12	[-0.26, 0.02]	0.0996	0.069	-0.01	[-0.49, 0.47]	0.9759	0.120
CVLT_8	0.05	[-0.11, 0.20]	0.5445	0.029	-0.23	[-0.62, 0.16]	0.2399	0.051
CVLT_9	0.06	[-0.84, 0.95]	0.9031	0.054	-0.29	[-1.75, 1.17]	0.6913	0.185
CVLT_10	0.13	[-0.04, 0.29]	0.1317	0.082	0.14	[-0.49, 0.78]	0.6544	0.011
CVLT_11	0.01	[-0.09, 0.11]	0.7898	0.012	0.25	[-0.04, 0.53]	0.0845	0.122
CVLT_12	0.01	[-0.06, 0.07]	0.8094	0.018	-0.39	[-0.68, -0.09]	0.0115	0.276
CVLT_13	0.02	[-0.01, 0.06]	0.2089	0.040	-0.53	[-1.00, -0.07]	0.0266	0.138
RPM	-0.69	[-1.53, 0.16]	0.1080	0.268	-0.02	[-1.41, 1.37]	0.9730	0.059
EHI	-0.78	[-3.75, 2.19]	0.6003	0.028	2.20	[-5.25, 9.66]	0.5534	0.060

Table S12. Male's Cognitive Test Results for Better and Worse Clusters, Model 1a: APOE e3e4 (significant p-values highlighted)

Variable	Better Cluster				Worse Cluster			
	Coef.	95% CI	*p*	R^2	Coef.	95% CI	*p*	R^2
CVLT_1	0.89	[-0.29, 2.07]	0.1348	0.110	-0.61	[-2.38, 1.15]	0.4862	0.014
CVLT_2	0.17	[-0.28, 0.61]	0.4520	0.046	0.09	[-0.30, 0.47]	0.6526	0.052
CVLT_3	0.17	[-0.13, 0.46]	0.2610	0.051	-0.29	[-0.79, 0.21]	0.2488	0.047
CVLT_4	0.49	[0.07, 0.92]	0.0243	0.143	0.11	[-0.31, 0.52]	0.6057	0.076
CVLT_5	0.40	[0.02, 0.79]	0.0420	0.131	-0.47	[-1.08, 0.15]	0.1321	0.060
CVLT_6	-0.11	[-0.37, 0.15]	0.4030	0.032	-0.27	[-0.76, 0.21]	0.2645	0.076
CVLT_7	0.03	[-0.21, 0.28]	0.7738	0.026	-0.34	[-0.94, 0.26]	0.2599	0.074
CVLT_8	-0.06	[-0.33, 0.21]	0.6509	0.033	-0.37	[-0.88, 0.14]	0.1489	0.130
CVLT_9	-0.26	[-0.70, 0.19]	0.2511	0.064	0.34	[-0.62, 1.29]	0.4836	0.051
CVLT_10	0.06	[-0.17, 0.30]	0.5918	0.011	0.06	[-0.38, 0.50]	0.7799	0.053
CVLT_11	0.15	[-0.08, 0.38]	0.1834	0.157	0.53	[-0.03, 1.09]	0.0643	0.127
CVLT_12	-0.06	[-0.17, 0.06]	0.3151	0.037	0.08	[-0.29, 0.45]	0.6570	0.050
CVLT_13	-0.02	[-0.11, 0.07]	0.6957	0.145	0.29	[-0.04, 0.62]	0.0878	0.160
RPM	-0.06	[-0.77, 0.64]	0.8553	0.027	-0.25	[-1.13, 0.63]	0.5746	0.173
EHI	-0.08	[-3.88, 3.72]	0.9656	0.088	1.75	[-3.74, 7.24]	0.5238	0.077

Table S13. Female's Cognitive Test Results for Better and Worse Clusters, Model 1b: APOE e3e4 (significant *p*-values highlighted)

Variable	Better Cluster				Worse Cluster			
	Coef.	95% CI	*p*	R^2	Coef.	95% CI	*p*	R^2
CVLT_1	0.41	[-5.07, 5.89]	0.8813	0.008	1.86	[-6.12, 9.85]	0.6388	0.030
CVLT_2	-0.56	[-2.52, 1.40]	0.5682	0.044	0.32	[-1.71, 2.36]	0.7505	0.086
CVLT_3	-0.03	[-1.09, 1.02]	0.9480	0.032	0.09	[-1.70, 1.89]	0.9156	0.014
CVLT_4	-1.68	[-3.88, 0.52]	0.1320	0.043	2.41	[0.31, 4.51]	0.0256	0.132
CVLT_5	0.21	[-0.85, 1.27]	0.6927	0.032	0.78	[-1.44, 3.01]	0.4789	0.057
CVLT_6	-0.09	[-0.97, 0.78]	0.8342	0.012	0.90	[-0.38, 2.18]	0.1629	0.109
CVLT_7	-0.16	[-0.99, 0.67]	0.6954	0.030	1.21	[-0.84, 3.26]	0.2403	0.066
CVLT_8	0.27	[-0.60, 1.14]	0.5422	0.033	-0.05	[-1.70, 1.60]	0.9523	0.022
CVLT_9	-1.67	[-6.73, 3.39]	0.5110	0.084	-1.76	[-6.44, 2.91]	0.4490	0.446
CVLT_10	0.15	[-0.83, 1.13]	0.7647	0.017	0.20	[-2.42, 2.82]	0.8781	0.027
CVLT_11	0.24	[-0.33, 0.80]	0.4028	0.027	-0.52	[-1.73, 0.69]	0.3873	0.039
CVLT_12	-0.16	[-0.54, 0.23]	0.4167	0.025	-0.54	[-1.45, 0.37]	0.2391	0.322
CVLT_13	0.02	[-0.20, 0.24]	0.8506	0.015	-2.17	[-3.60, -0.74]	0.0040	0.228
RPM	-0.08	[-5.72, 5.57]	0.9785	0.010	3.91	[-1.17, 9.00]	0.1271	0.268
EHI	3.80	[-12.57, 20.17]	0.6434	0.030	16.14	[-14.32, 46.60]	0.2897	0.061

Table S14. Male's Cognitive Test Results for Better and Worse Clusters, Model 1b: APOE e3e4 (significant p-values highlighted)

Variable	Better Cluster				Worse Cluster			
	Coef.	95% CI	*p*	R^2	Coef.	95% CI	*p*	R^2
CVLT_1	0.05	[-7.49, 7.60]	0.9883	0.048	-1.81	[-8.55, 4.92]	0.5892	0.036
CVLT_2	-0.47	[-3.13, 2.19]	0.7222	0.103	-0.67	[-2.24, 0.89]	0.3877	0.063
CVLT_3	-0.09	[-1.98, 1.79]	0.9206	0.038	0.52	[-1.50, 2.53]	0.6077	0.026
CVLT_4	-1.47	[-4.21, 1.27]	0.2821	0.103	0.79	[-0.80, 2.39]	0.3205	0.083
CVLT_5	0.36	[-1.81, 2.52]	0.7379	0.101	0.33	[-2.21, 2.87]	0.7942	0.028
CVLT_6	0.30	[-1.19, 1.79]	0.6839	0.165	-0.20	[-2.13, 1.73]	0.8371	0.004
CVLT_7	0.46	[-0.92, 1.84]	0.5008	0.182	0.61	[-1.66, 2.87]	0.5898	0.072
CVLT_8	0.47	[-1.16, 2.10]	0.5628	0.166	-0.84	[-2.93, 1.25]	0.4209	0.063
CVLT_9	-2.74	[-5.39, -0.08]	0.0441	0.144	-3.26	[-6.83, 0.31]	0.0722	0.092
CVLT_10	0.37	[-1.11, 1.84]	0.6169	0.014	0.27	[-1.69, 2.24]	0.7824	0.009
CVLT_11	-0.08	[-1.03, 0.87]	0.8647	0.027	0.09	[-2.50, 2.69]	0.9420	0.048
CVLT_12	-0.04	[-0.73, 0.64]	0.8974	0.025	-0.51	[-1.91, 0.88]	0.4622	0.037
CVLT_13	-0.07	[-0.64, 0.49]	0.7908	0.023	0.47	[-1.08, 2.03]	0.5405	0.071
RPM	1.19	[-3.14, 5.52]	0.5783	0.088	-4.13	[-7.41, -0.84]	0.0151	0.159
EHI	6.38	[-16.57, 29.33]	0.5744	0.139	1.22	[-21.21, 23.66]	0.9128	0.088

Table S15. Female's Cognitive Test Results for Better and Worse Clusters, Model 1c: APOE e3e4 (significant p-values highlighted)

Variable	Better Cluster				Worse Cluster			
	Coef.	95% CI	*p*	R^2	Coef.	95% CI	*p*	R^2
CVLT_1	-0.15	[-0.48, 0.17]	0.3516	0.037	-0.49	[-6.15, 5.17]	0.8608	0.156
CVLT_2	-0.07	[-0.19, 0.05]	0.2270	0.075	-0.29	[-1.81, 1.22]	0.6962	0.122
CVLT_3	0.02	[-0.04, 0.08]	0.5478	0.065	-0.47	[-1.72, 0.79]	0.4562	0.166
CVLT_4	-0.06	[-0.19, 0.07]	0.3332	0.071	-0.97	[-2.50, 0.56]	0.2058	0.201
CVLT_5	-0.07	[-0.12, -0.01]	0.0315	0.160	-0.44	[-1.92, 1.05]	0.5534	0.273
CVLT_6	0.00	[-0.05, 0.05]	0.9763	0.080	-0.66	[-1.58, 0.27]	0.1567	0.200
CVLT_7	-0.04	[-0.09, 0.01]	0.1047	0.098	-1.20	[-2.56, 0.15]	0.0804	0.291
CVLT_8	0.02	[-0.03, 0.07]	0.4741	0.055	-0.27	[-1.44, 0.91]	0.6441	0.139
CVLT_9	0.02	[-0.28, 0.32]	0.9031	0.094	-4.06	[-7.26, -0.87]	0.0144	0.550
CVLT_10	0.04	[-0.02, 0.10]	0.1588	0.106	-0.80	[-2.71, 1.12]	0.4028	0.095
CVLT_11	0.01	[-0.03, 0.04]	0.7276	0.039	-0.67	[-1.50, 0.15]	0.1044	0.231
CVLT_12	0.00	[-0.02, 0.02]	0.9687	0.179	-0.59	[-1.14, -0.04]	0.0352	0.583
CVLT_13	0.01	[-0.01, 0.02]	0.2589	0.043	0.46	[-0.56, 1.49]	0.3609	0.321
RPM	-0.19	[-0.48, 0.09]	0.1811	0.291	-0.54	[-4.06, 2.98]	0.7565	0.393
EHI	-0.15	[-1.12, 0.83]	0.7603	0.046	-15.00	[-36.72, 6.73]	0.1693	0.172

Table S16. Male's Cognitive Test Results for Better and Worse Clusters, Model 1c: APOE e3e4 (significant p-values highlighted)

**Variable**	**Better Cluster**				**Worse Cluster**			
	Coef.	95% CI	*p*	R^2	Coef.	95% CI	*p*	R^2
CVLT_1	0.22	[-0.22, 0.66]	0.3129	0.166	-2.42	[-4.87, 0.03]	0.0526	0.174
CVLT_2	0.02	[-0.15, 0.18]	0.8153	0.120	-0.17	[-0.77, 0.43]	0.5738	0.098
CVLT_3	0.05	[-0.06, 0.17]	0.3311	0.105	-0.55	[-1.30, 0.20]	0.1428	0.133
CVLT_4	0.11	[-0.06, 0.27]	0.1896	0.200	-0.24	[-0.84, 0.37]	0.4327	0.150
CVLT_5	0.11	[-0.01, 0.23]	0.0773	0.277	-1.16	[-2.02, -0.30]	0.0098	0.273
CVLT_6	-0.00	[-0.09, 0.09]	0.9667	0.182	-0.87	[-1.52, -0.22]	0.0100	0.270
CVLT_7	0.03	[-0.06, 0.11]	0.4872	0.213	-1.13	[-1.87, -0.39]	0.0037	0.364
CVLT_8	0.01	[-0.09, 0.11]	0.9045	0.183	-0.89	[-1.57, -0.21]	0.0116	0.361
CVLT_9	-0.12	[-0.28, 0.04]	0.1362	0.223	-1.72	[-2.92, -0.53]	0.0059	0.343
CVLT_10	0.07	[-0.02, 0.15]	0.1348	0.124	0.10	[-0.63, 0.84]	0.7812	0.104
CVLT_11	0.01	[-0.05, 0.06]	0.8431	0.058	0.79	[-0.13, 1.70]	0.0895	0.240
CVLT_12	-0.01	[-0.05, 0.03]	0.6299	0.057	-0.08	[-0.63, 0.46]	0.7594	0.053
CVLT_13	-0.01	[-0.05, 0.02]	0.3819	0.159	0.59	[0.06, 1.11]	0.0310	0.305
RPM	-0.02	[-0.28, 0.24]	0.8817	0.145	-0.36	[-1.54, 0.83]	0.5461	0.293
EHI	-0.14	[-1.29, 1.01]	0.8062	0.442	1.75	[-7.01, 10.52]	0.6873	0.099

Table S17. Female's Cognitive Test Results for Better and Worse Clusters, Model 2: PICALM_GG (significant *p*-values highlighted)

**Variable**	**Better Cluster**				**Worse Cluster**			
	Coef.	95% CI	*p*	R^2	Coef.	95% CI	*p*	R^2
CVLT_1	-1.60	[-4.02, 0.82]	0.1903	0.029	0.16	[-4.30, 4.63]	0.9424	0.000
CVLT_2	-0.51	[-1.40, 0.39]	0.2638	0.022	0.16	[-0.98, 1.29]	0.7823	0.002
CVLT_3	-0.07	[-0.57, 0.43]	0.7875	0.001	-0.15	[-1.11, 0.81]	0.7550	0.002
CVLT_4	0.06	[-0.99, 1.10]	0.9130	0.000	0.64	[-0.56, 1.85]	0.2843	0.028
CVLT_5	-0.20	[-0.68, 0.29]	0.4272	0.011	0.13	[-1.10, 1.37]	0.8280	0.001
CVLT_6	0.17	[-0.24, 0.57]	0.4136	0.012	0.30	[-0.43, 1.04]	0.4084	0.017
CVLT_7	-0.02	[-0.40, 0.35]	0.9002	0.000	0.05	[-1.08, 1.19]	0.9268	0.000
CVLT_8	0.17	[-0.23, 0.57]	0.3997	0.012	0.02	[-0.88, 0.91]	0.9720	0.000
CVLT_9	0.98	[-1.33, 3.30]	0.3993	0.012	-0.25	[-3.83, 3.33]	0.8891	0.001
CVLT_10	-0.13	[-0.57, 0.30]	0.5483	0.006	-2.17	[-3.41, -0.92]	0.0011	0.232
CVLT_11	-0.09	[-0.34, 0.16]	0.4540	0.010	-0.84	[-1.46, -0.23]	0.0084	0.158
CVLT_12	0.08	[-0.09, 0.25]	0.3611	0.014	0.09	[-0.51, 0.68]	0.7715	0.002
CVLT_13	0.07	[-0.02, 0.17]	0.1156	0.042	-0.41	[-1.28, 0.45]	0.3403	0.023
RPM	-0.38	[-2.86, 2.09]	0.7575	0.002	0.50	[-2.67, 3.67]	0.7519	0.003
EHI	1.31	[-6.28, 8.90]	0.7311	0.002	12.01	[-4.62, 28.64]	0.1522	0.049

Table S18. Male's Cognitive Test Results for Better and Worse Clusters, Model 2: PICALM_GG (significant *p*-values highlighted)

Variable	Better Cluster				Worse Cluster			
	Coef.	95% CI	*p*	R^2	Coef.	95% CI	*p*	R^2
CVLT_1	0.27	[-3.23, 3.76]	0.8777	0.001	2.38	[-1.82, 6.57]	0.2604	0.026
CVLT_2	0.47	[-0.79, 1.73]	0.4520	0.016	0.03	[-0.91, 0.97]	0.9443	0.000
CVLT_3	0.00	[-0.85, 0.85]	1.0000	0.000	0.84	[-0.36, 2.03]	0.1653	0.039
CVLT_4	0.00	[-1.28, 1.28]	1.0000	0.000	0.31	[-0.73, 1.34]	0.5544	0.007
CVLT_5	-0.04	[-1.19, 1.11]	0.9446	0.000	0.60	[-0.91, 2.11]	0.4283	0.013
CVLT_6	-0.11	[-0.84, 0.62]	0.7665	0.003	0.30	[-0.90, 1.50]	0.6195	0.005
CVLT_7	0.26	[-0.43, 0.94]	0.4520	0.016	0.47	[-1.02, 1.96]	0.5294	0.008
CVLT_8	0.10	[-0.66, 0.86]	0.7979	0.002	0.01	[-1.30, 1.32]	0.9900	0.000
CVLT_9	-0.78	[-2.03, 0.47]	0.2158	0.042	-0.32	[-2.67, 2.03]	0.7872	0.002
CVLT_10	0.22	[-0.44, 0.89]	0.5029	0.013	0.48	[-0.59, 1.55]	0.3710	0.016
CVLT_11	-0.20	[-0.89, 0.50]	0.5647	0.009	0.89	[-0.52, 2.30]	0.2121	0.032
CVLT_12	0.04	[-0.28, 0.36]	0.8024	0.002	0.61	[-0.28, 1.49]	0.1765	0.037
CVLT_13	0.10	[-0.18, 0.37]	0.4852	0.014	0.51	[-0.34, 1.37]	0.2312	0.029
RPM	-0.13	[-2.12, 1.86]	0.8948	0.001	0.40	[-1.91, 2.71]	0.7307	0.002
EHI	-4.49	[-15.48, 6.51]	0.4132	0.019	6.31	[-7.23, 19.85]	0.3537	0.018

Table S19. Female's Cognitive Test Results for Better and Worse Clusters, Model 2a: PICALM_GG (significant *p*-values highlighted)

Variable	Better Cluster				Worse Cluster			
	Coef.	95% CI	*p*	R^2	Coef.	95% CI	*p*	R^2
CVLT_1	-0.66	[-1.48, 0.16]	0.1134	0.077	-0.25	[-1.64, 1.14]	0.7162	0.059
CVLT_2	-0.33	[-0.63, -0.03]	0.0323	0.101	-0.23	[-0.59, 0.12]	0.1838	0.068
CVLT_3	-0.05	[-0.22, 0.12]	0.5919	0.047	-0.18	[-0.47, 0.11]	0.2233	0.092
CVLT_4	-0.18	[-0.54, 0.18]	0.3296	0.026	-0.11	[-0.49, 0.26]	0.5402	0.082
CVLT_5	-0.18	[-0.35, -0.02]	0.0266	0.101	-0.04	[-0.42, 0.35]	0.8523	0.065
CVLT_6	-0.06	[-0.20, 0.07]	0.3427	0.085	0.08	[-0.15, 0.31]	0.4883	0.045
CVLT_7	-0.07	[-0.19, 0.06]	0.3091	0.037	0.07	[-0.27, 0.41]	0.6818	0.126
CVLT_8	-0.08	[-0.22, 0.06]	0.2393	0.053	0.16	[-0.12, 0.44]	0.2556	0.036
CVLT_9	0.25	[-0.55, 1.05]	0.5382	0.034	0.05	[-1.08, 1.19]	0.9232	0.023
CVLT_10	0.08	[-0.06, 0.23]	0.2608	0.071	0.12	[-0.27, 0.51]	0.5386	0.266
CVLT_11	0.02	[-0.06, 0.11]	0.5807	0.026	0.08	[-0.10, 0.27]	0.3680	0.258
CVLT_12	0.01	[-0.05, 0.07]	0.7843	0.032	0.02	[-0.16, 0.21]	0.7978	0.054
CVLT_13	0.00	[-0.03, 0.04]	0.8371	0.043	-0.02	[-0.30, 0.25]	0.8618	0.034
RPM	-0.72	[-1.45, 0.02]	0.0547	0.278	0.65	[-0.32, 1.61]	0.1833	0.100
EHI	2.19	[-0.38, 4.77]	0.0937	0.053	1.86	[-3.19, 6.91]	0.4611	0.146

Table S20. Male's Cognitive Test Results for Better and Worse Clusters, Model 2a: PICALM_GG (significant p-values highlighted)

Variable	Better Cluster				Worse Cluster			
	Coef.	95% CI	p	R^2	Coef.	95% CI	p	R^2
CVLT_1	-0.14	[-1.22, 0.95]	0.7960	0.035	-0.63	[-2.18, 0.93]	0.4200	0.041
CVLT_2	0.18	[-0.21, 0.58]	0.3472	0.042	0.15	[-0.20, 0.49]	0.4009	0.024
CVLT_3	0.05	[-0.22, 0.31]	0.7314	0.016	-0.46	[-0.89, -0.04]	0.0338	0.131
CVLT_4	-0.28	[-0.67, 0.12]	0.1615	0.058	0.11	[-0.26, 0.49]	0.5533	0.058
CVLT_5	-0.22	[-0.58, 0.13]	0.2087	0.063	-0.18	[-0.74, 0.38]	0.5206	0.028
CVLT_6	0.01	[-0.22, 0.24]	0.9121	0.016	-0.31	[-0.74, 0.12]	0.1591	0.089
CVLT_7	0.05	[-0.17, 0.26]	0.6688	0.030	-0.25	[-0.79, 0.30]	0.3660	0.054
CVLT_8	-0.02	[-0.26, 0.22]	0.8955	0.005	-0.20	[-0.67, 0.26]	0.3866	0.099
CVLT_9	0.28	[-0.10, 0.66]	0.1439	0.119	-0.02	[-0.88, 0.85]	0.9688	0.033
CVLT_10	0.05	[-0.16, 0.26]	0.6236	0.021	0.08	[-0.30, 0.47]	0.6623	0.076
CVLT_11	0.02	[-0.19, 0.23]	0.8461	0.070	0.47	[-0.03, 0.96]	0.0636	0.142
CVLT_12	0.01	[-0.09, 0.12]	0.7778	0.005	-0.15	[-0.48, 0.17]	0.3504	0.071
CVLT_13	0.08	[0.01, 0.16]	0.0312	0.270	0.22	[-0.08, 0.52]	0.1545	0.128
RPM	0.32	[-0.29, 0.93]	0.2946	0.051	0.37	[-0.46, 1.19]	0.3777	0.083
EHI	-1.07	[-4.45, 2.31]	0.5246	0.071	0.59	[-4.47, 5.64]	0.8160	0.019

Table S21. Female's Cognitive Test Results for Better and Worse Clusters, Model 2b: PICALM_GG (significant p-values highlighted)

Variable	Better Cluster				Worse Cluster			
	Coef.	95% CI	p	R^2	Coef.	95% CI	p	R^2
CVLT_1	-3.71	[-7.92, 0.49]	0.0823	0.074	-2.00	[-8.71, 4.70]	0.5485	0.035
CVLT_2	-0.82	[-2.38, 0.74]	0.2951	0.035	-0.99	[-2.67, 0.69]	0.2405	0.117
CVLT_3	-0.17	[-1.02, 0.67]	0.6800	0.024	0.36	[-1.16, 1.88]	0.6337	0.011
CVLT_4	-1.14	[-2.89, 0.62]	0.1992	0.039	-0.31	[-2.18, 1.56]	0.7405	0.026
CVLT_5	-0.58	[-1.40, 0.25]	0.1655	0.074	-0.67	[-2.56, 1.22]	0.4774	0.037
CVLT_6	-0.32	[-1.01, 0.37]	0.3536	0.025	-0.31	[-1.41, 0.80]	0.5789	0.067
CVLT_7	-0.35	[-1.01, 0.30]	0.2858	0.036	-0.03	[-1.81, 1.76]	0.9760	0.003
CVLT_8	-0.34	[-1.03, 0.34]	0.3201	0.056	0.48	[-0.91, 1.87]	0.4892	0.019
CVLT_9	1.40	[-2.71, 5.51]	0.4980	0.042	3.26	[-1.36, 7.89]	0.1612	0.236
CVLT_10	0.13	[-0.65, 0.91]	0.7375	0.023	0.60	[-1.31, 2.51]	0.5278	0.271
CVLT_11	0.26	[-0.19, 0.70]	0.2562	0.044	0.29	[-0.66, 1.24]	0.5388	0.172
CVLT_12	-0.14	[-0.44, 0.16]	0.3559	0.038	-0.23	[-1.08, 0.62]	0.5867	0.169
CVLT_13	0.04	[-0.12, 0.21]	0.6054	0.054	0.85	[-0.45, 2.15]	0.1935	0.091
RPM	-1.02	[-5.51, 3.46]	0.6490	0.011	-5.37	[-9.41, -1.32]	0.0108	0.347
EHI	1.15	[-12.04, 14.34]	0.8621	0.003	-1.01	[-26.47, 24.46]	0.9365	0.075

Table S22. Male's Cognitive Test Results for Better and Worse Clusters, Model 2b: PICALM_GG (significant *p*-values highlighted)

Variable	Better Cluster				Worse Cluster			
	Coef.	95% CI	*p*	R^2	Coef.	95% CI	*p*	R^2
CVLT_1	1.98	[-5.59, 9.55]	0.5965	0.019	0.25	[-5.87, 6.37]	0.9345	0.031
CVLT_2	1.32	[-1.30, 3.94]	0.3120	0.111	-0.09	[-1.54, 1.35]	0.8958	0.028
CVLT_3	0.70	[-1.16, 2.56]	0.4498	0.038	-0.38	[-2.18, 1.43]	0.6760	0.050
CVLT_4	2.02	[-0.64, 4.68]	0.1313	0.135	-0.54	[-2.04, 0.96]	0.4722	0.016
CVLT_5	1.49	[-0.55, 3.53]	0.1463	0.182	-0.47	[-2.76, 1.83]	0.6833	0.029
CVLT_6	0.12	[-1.36, 1.60]	0.8686	0.154	-0.80	[-2.53, 0.93]	0.3575	0.025
CVLT_7	0.40	[-0.98, 1.77]	0.5610	0.172	-0.28	[-2.38, 1.83]	0.7927	0.021
CVLT_8	0.23	[-1.42, 1.88]	0.7770	0.126	-1.28	[-3.18, 0.62]	0.1814	0.055
CVLT_9	1.00	[-1.74, 3.74]	0.4619	0.069	3.67	[0.50, 6.84]	0.0243	0.129
CVLT_10	0.36	[-1.10, 1.82]	0.6208	0.014	-0.35	[-2.12, 1.42]	0.6901	0.020
CVLT_11	-0.08	[-1.02, 0.87]	0.8688	0.027	-0.42	[-2.76, 1.93]	0.7214	0.055
CVLT_12	0.05	[-0.63, 0.74]	0.8702	0.018	-0.34	[-1.60, 0.92]	0.5882	0.038
CVLT_13	0.04	[-0.52, 0.61]	0.8741	0.021	0.65	[-0.79, 2.08]	0.3671	0.041
RPM	0.49	[-3.80, 4.79]	0.8155	0.081	-0.29	[-3.54, 2.95]	0.8555	0.002
EHI	-33.90	[-52.43, -15.37]	0.0008	0.426	8.24	[-12.48, 28.97]	0.4261	0.051

Table S23. Female's Cognitive Test Results for Better and Worse Clusters, Model 2c: PICALM_GG (significant *p*-values highlighted)

Variable	Better Cluster				Worse Cluster			
	Coef.	95% CI	*p*	R^2	Coef.	95% CI	*p*	R^2
CVLT_1	2.04	[-4.77, 8.86]	0.5495	0.109	0.96	[-0.99, 2.91]	0.3248	0.176
CVLT_2	1.37	[-1.08, 3.81]	0.2682	0.130	0.12	[-0.38, 0.63]	0.6281	0.195
CVLT_3	0.03	[-1.35, 1.41]	0.9631	0.050	0.24	[-0.19, 0.68]	0.2625	0.171
CVLT_4	-0.28	[-3.10, 2.54]	0.8413	0.086	0.56	[0.03, 1.09]	0.0381	0.208
CVLT_5	0.02	[-1.29, 1.32]	0.9795	0.156	0.24	[-0.30, 0.77]	0.3758	0.224
CVLT_6	0.51	[-0.57, 1.58]	0.3478	0.138	0.20	[-0.14, 0.53]	0.2406	0.146
CVLT_7	-0.08	[-1.14, 0.99]	0.8842	0.073	0.23	[-0.27, 0.74]	0.3516	0.197
CVLT_8	-0.02	[-1.13, 1.09]	0.9705	0.094	0.33	[-0.07, 0.72]	0.0996	0.208
CVLT_9	0.12	[-6.64, 6.88]	0.9717	0.050	0.71	[-0.67, 2.09]	0.3039	0.307
CVLT_10	0.13	[-1.10, 1.36]	0.8284	0.104	-0.43	[-0.96, 0.10]	0.1097	0.426
CVLT_11	0.01	[-0.72, 0.74]	0.9814	0.061	-0.13	[-0.40, 0.15]	0.3520	0.302
CVLT_12	-0.09	[-0.55, 0.37]	0.6990	0.180	0.11	[-0.15, 0.36]	0.3959	0.245
CVLT_13	0.00	[-0.28, 0.28]	0.9936	0.054	-0.07	[-0.47, 0.34]	0.7410	0.109
RPM	0.15	[-6.01, 6.32]	0.9602	0.315	-0.22	[-1.36, 0.92]	0.6987	0.476
EHI	-9.51	[-30.24, 11.23]	0.3614	0.098	-5.05	[-12.28, 2.18]	0.1643	0.246

Table S24. Male's Cognitive Test Results for Better and Worse Clusters, Model 2c: PICALM_GG (significant *p*-values highlighted)

Variable	Better Cluster				Worse Cluster			
	Coef.	95% CI	*p*	R^2	Coef.	95% CI	*p*	R^2
CVLT_1	0.02	[-0.83, 0.86]	0.9689	0.085	0.60	[-2.27, 3.46]	0.6753	0.123
CVLT_2	0.17	[-0.12, 0.45]	0.2445	0.193	-0.11	[-0.81, 0.58]	0.7440	0.067
CVLT_3	-0.02	[-0.24, 0.19]	0.8141	0.059	0.17	[-0.63, 0.98]	0.6652	0.216
CVLT_4	-0.10	[-0.40, 0.20]	0.5152	0.170	-0.11	[-0.82, 0.61]	0.7609	0.070
CVLT_5	-0.22	[-0.44, -0.01]	0.0400	0.326	0.62	[-0.44, 1.68]	0.2422	0.145
CVLT_6	-0.09	[-0.25, 0.08]	0.2987	0.197	0.45	[-0.30, 1.21]	0.2292	0.236
CVLT_7	-0.07	[-0.22, 0.09]	0.3830	0.226	0.05	[-0.91, 1.01]	0.9147	0.155
CVLT_8	-0.10	[-0.29, 0.08]	0.2578	0.186	0.37	[-0.45, 1.19]	0.3691	0.273
CVLT_9	0.34	[0.06, 0.62]	0.0194	0.269	1.29	[-0.14, 2.72]	0.0757	0.265
CVLT_10	-0.08	[-0.25, 0.08]	0.2968	0.090	-0.19	[-1.00, 0.62]	0.6351	0.158
CVLT_11	0.06	[-0.04, 0.16]	0.2422	0.161	0.12	[-0.96, 1.21]	0.8176	0.163
CVLT_12	-0.01	[-0.09, 0.07]	0.8403	0.042	0.17	[-0.43, 0.76]	0.5789	0.105
CVLT_13	0.04	[-0.01, 0.09]	0.1406	0.346	0.04	[-0.61, 0.68]	0.9125	0.188
RPM	-0.09	[-0.56, 0.39]	0.7096	0.159	0.38	[-1.07, 1.83]	0.5994	0.174
EHI	1.72	[-0.15, 3.59]	0.0698	0.561	-1.22	[-11.38, 8.94]	0.8084	0.056

1.2 Full Output of ANOVA and Tukey HSD Tests

Table S25. Multiple Comparison of Means - Tukey HSD for Variable: `CVLT_1`
ANOVA p-value: 1.824×10^{-34}

Group 1	Group 2	Mean Diff.	p-adj	Lower	Upper	Reject
female_better	female_worse	-11.33	0.0000	-14.48	-8.18	True
female_better	male_better	-3.43	0.0360	-6.69	-0.16	True
female_better	male_worse	-17.07	0.0000	-20.08	-14.07	True
female_worse	male_better	7.90	0.0000	4.39	11.41	True
female_worse	male_worse	-5.75	0.0001	-9.01	-2.48	True
male_better	male_worse	-13.65	0.0000	-17.03	-10.27	True

Group	Mean	STD
female_better	69.583	4.681
female_worse	58.256	7.004
male_better	66.158	5.175
male_worse	52.510	7.223

Table S26. Multiple Comparison of Means - Tukey HSD for Variable: `CVLT_2`
ANOVA p-value: 4.888×10^{-13}

Group 1	Group 2	Mean Diff.	p-adj	Lower	Upper	Reject
female_better	female_worse	-1.81	0.0000	-2.71	-0.91	True
female_better	male_better	-1.04	0.0217	-1.98	-0.11	True
female_better	male_worse	-2.70	0.0000	-3.55	-1.84	True
female_worse	male_better	0.77	0.1990	-0.24	1.77	False
female_worse	male_worse	-0.89	0.0692	-1.82	0.05	False
male_better	male_worse	-1.65	0.0001	-2.62	-0.69	True

Group	Mean	STD
female_better	10.833	1.729
female_worse	9.023	1.779
male_better	9.789	1.877
male_worse	8.137	1.600

Table S27. Multiple Comparison of Means - Tukey HSD for Variable: CVLT_3
ANOVA p-value: 4.854×10^{-27}

Group 1	Group 2	Mean Diff.	p-adj	Lower	Upper	Reject
female_better	female_worse	-2.01	0.0000	-2.78	-1.23	True
female_better	male_better	-0.33	0.7060	-1.14	0.47	False
female_better	male_worse	-3.49	0.0000	-4.23	-2.75	True
female_worse	male_better	1.67	0.0000	0.81	2.54	True
female_worse	male_worse	-1.48	0.0000	-2.29	-0.68	True
male_better	male_worse	-3.16	0.0000	-3.99	-2.33	True

Group	Mean	STD
female_better	15.333	0.951
female_worse	13.326	1.507
male_better	15.000	1.252
male_worse	11.843	2.072

Table S28. Multiple Comparison of Means - Tukey HSD for Variable: CVLT_4
ANOVA p-value: 4.364×10^{-6}

Group 1	Group 2	Mean Diff.	p-adj	Lower	Upper	Reject
female_better	female_worse	-0.98	0.0518	-1.96	0.01	False
female_better	male_better	-0.62	0.3993	-1.64	0.40	False
female_better	male_worse	-1.94	0.0000	-2.88	-1.00	True
female_worse	male_better	0.36	0.8286	-0.73	1.46	False
female_worse	male_worse	-0.96	0.0710	-1.98	0.05	False
male_better	male_worse	-1.32	0.0073	-2.38	-0.27	True

Group	Mean	STD
female_better	9.117	1.992
female_worse	8.140	1.910
male_better	8.500	1.900
male_worse	7.176	1.763

Table S29. Multiple Comparison of Means - Tukey HSD for Variable: CVLT_5
ANOVA p-value: 6.921×10^{-31}

Group 1	Group 2	Mean Diff.	p-adj	Lower	Upper	Reject
female_better	female_worse	-3.17	0.0000	-4.14	-2.21	True
female_better	male_better	-1.32	0.0041	-2.32	-0.32	True
female_better	male_worse	-4.94	0.0000	-5.85	-4.02	True
female_worse	male_better	1.85	0.0001	0.78	2.92	True
female_worse	male_worse	-1.76	0.0000	-2.76	-0.77	True
male_better	male_worse	-3.61	0.0000	-4.64	-2.58	True

Group	Mean	STD
female_better	15.033	0.938
female_worse	11.860	1.934
male_better	13.711	1.707
male_worse	10.098	2.579

Table S30. Multiple Comparison of Means - Tukey HSD for Variable: CVLT_6
ANOVA p-value: 3.421×10^{-31}

Group 1	Group 2	Mean Diff.	p-adj	Lower	Upper	Reject
female_better	female_worse	-2.40	0.0000	-3.10	-1.70	True
female_better	male_better	-0.48	0.3164	-1.21	0.25	False
female_better	male_worse	-3.43	0.0000	-4.10	-2.77	True
female_worse	male_better	1.92	0.0000	1.14	2.70	True
female_worse	male_worse	-1.03	0.0017	-1.76	-0.30	True
male_better	male_worse	-2.95	0.0000	-3.70	-2.20	True

Group	Mean	STD
female_better	14.983	0.770
female_worse	12.581	1.159
male_better	14.500	1.084
male_worse	11.549	2.052

Table S31. Multiple Comparison of Means - Tukey HSD for Variable: CVLT_7
ANOVA p-value: 1.735×10^{-34}

Group 1	Group 2	Mean Diff.	p-adj	Lower	Upper	Reject
female_better	female_worse	-2.71	0.0000	-3.57	-1.84	True
female_better	male_better	-0.59	0.3188	-1.49	0.30	False
female_better	male_worse	-4.68	0.0000	-5.50	-3.85	True
female_worse	male_better	2.12	0.0000	1.15	3.08	True
female_worse	male_worse	-1.97	0.0000	-2.87	-1.07	True
male_better	male_worse	-4.08	0.0000	-5.01	-3.16	True

Group	Mean	STD
female_better	15.383	0.715
female_worse	12.674	1.782
male_better	14.789	1.018
male_worse	10.706	2.540

Table S32. Multiple Comparison of Means - Tukey HSD for Variable: CVLT_8
ANOVA p-value: 1.282×10^{-26}

Group 1	Group 2	Mean Diff.	p-adj	Lower	Upper	Reject
female_better	female_worse	-2.35	0.0000	-3.12	-1.59	True
female_better	male_better	-0.54	0.3046	-1.33	0.26	False
female_better	male_worse	-3.38	0.0000	-4.11	-2.65	True
female_worse	male_better	1.82	0.0000	0.96	2.67	True
female_worse	male_worse	-1.03	0.0052	-1.82	-0.23	True
male_better	male_worse	-2.85	0.0000	-3.67	-2.02	True

Group	Mean	STD
female_better	15.167	0.763
female_worse	12.814	1.402
male_better	14.632	1.125
male_worse	11.784	2.230

Table S33. Multiple Comparison of Means - Tukey HSD for Variable: CVLT_9
ANOVA p-value: 4.752×10^{-5}

Group 1	Group 2	Mean Diff.	p-adj	Lower	Upper	Reject
female_better	female_worse	2.68	0.0106	0.46	4.89	True
female_better	male_better	-1.77	0.1919	-4.07	0.53	False
female_better	male_worse	1.19	0.4604	-0.92	3.30	False
female_worse	male_better	-4.45	0.0000	-6.91	-1.98	True
female_worse	male_worse	-1.48	0.3381	-3.78	0.81	False
male_better	male_worse	2.96	0.0077	0.59	5.33	True

Group	Mean	STD
female_better	3.533	4.451
female_worse	6.209	5.621
male_better	1.763	1.895
male_worse	4.725	4.000

Table S34. Multiple Comparison of Means - Tukey HSD for Variable: CVLT_10
ANOVA p-value: 1.958×10^{-5}

Group 1	Group 2	Mean Diff.	p-adj	Lower	Upper	Reject
female_better	female_worse	1.09	0.0032	0.28	1.89	True
female_better	male_better	0.17	0.9541	-0.67	1.00	False
female_better	male_worse	1.29	0.0001	0.52	2.05	True
female_worse	male_better	-0.92	0.0417	-1.82	-0.02	True
female_worse	male_worse	0.20	0.9257	-0.63	1.03	False
male_better	male_worse	1.12	0.0051	0.26	1.98	True

Group	Mean	STD
female_better	0.517	0.833
female_worse	1.605	2.227
male_better	0.684	0.989
male_worse	1.804	1.833

Table S35. Multiple Comparison of Means - Tukey HSD for Variable: CVLT_11
ANOVA p-value: 3.418×10^{-5}

Group 1	Group 2	Mean Diff.	p-adj	Lower	Upper	Reject
female_better	female_worse	0.54	0.2414	-0.21	1.30	False
female_better	male_better	0.35	0.6451	-0.43	1.13	False
female_better	male_worse	1.35	0.0000	0.63	2.07	True
female_worse	male_better	-0.19	0.9341	-1.03	0.65	False
female_worse	male_worse	0.80	0.0398	0.03	1.58	True
male_better	male_worse	1.00	0.0086	0.19	1.80	True

Group	Mean	STD
female_better	0.200	0.480
female_worse	0.744	1.049
male_better	0.553	1.032
male_worse	1.549	2.436

Table S36. Multiple Comparison of Means - Tukey HSD for Variable: `CVLT_12`
ANOVA p-value: 3.93×10^{-10}

Group 1	Group 2	Mean Diff.	p-adj	Lower	Upper	Reject
female_better	female_worse	nan	nan	nan	nan	False
female_better	male_better	-0.09	nan	nan	nan	False
female_better	male_worse	-1.16	nan	nan	nan	False
female_worse	male_better	nan	nan	nan	nan	False
female_worse	male_worse	nan	nan	nan	nan	False
male_better	male_worse	-1.06	nan	nan	nan	False

Group	Mean	STD
female_better	15.883	0.324
female_worse	15.071	0.921
male_better	15.789	0.474
male_worse	14.725	1.537

Table S37. Multiple Comparison of Means - Tukey HSD for Variable: `CVLT_13`
ANOVA p-value: 3.439×10^{-8}

Group 1	Group 2	Mean Diff.	p-adj	Lower	Upper	Reject
female_better	female_worse	nan	nan	nan	nan	False
female_better	male_better	0.10	nan	nan	nan	False
female_better	male_worse	1.06	nan	nan	nan	False
female_worse	male_better	nan	nan	nan	nan	False
female_worse	male_worse	nan	nan	nan	nan	False
male_better	male_worse	0.97	nan	nan	nan	False

Group	Mean	STD
female_better	0.033	0.181
female_worse	0.881	1.347
male_better	0.132	0.414
male_worse	1.098	1.473

Table S38. Multiple Comparison of Means - Tukey HSD for Variable: `RPM`
ANOVA p-value: 1.944×10^{-5}

Group 1	Group 2	Mean Diff.	p-adj	Lower	Upper	Reject
female_better	female_worse	-2.736	0.0089	-4.957	-0.514	True
female_better	male_better	1.830	0.171	-0.475	4.135	False
female_better	male_worse	-1.473	0.2754	-3.590	0.645	False
female_worse	male_better	4.566	0.0	2.090	7.041	True
female_worse	male_worse	1.263	0.487	-1.039	3.565	False
male_better	male_worse	-3.302	0.0023	-5.685	-0.920	True

Group	Mean	STD
female_better	53.433	4.731
female_worse	50.698	4.983
male_better	55.263	2.947
male_worse	51.961	3.934

Table S39. Multiple Comparison of Means - Tukey HSD for Variable: EHI
ANOVA p-value: 0.0007068

Group 1	Group 2	Mean Diff.	p-adj	Lower	Upper	Reject
female_better	female_worse	-15.542	0.0012	-26.191	-4.893	True
female_better	male_better	-0.176	1.0	-11.226	10.873	False
female_better	male_worse	-7.820	0.1928	-17.971	2.331	False
female_worse	male_better	15.365	0.0052	3.499	27.232	True
female_worse	male_worse	7.722	0.27	-3.313	18.756	False
male_better	male_worse	-7.644	0.3086	-19.065	3.778	False

Group	Mean	STD
female_better	89.650	14.505
female_worse	74.108	26.754
male_better	89.474	16.431
male_worse	81.830	23.241

1.3 Box-plot summary of clasters comparisons

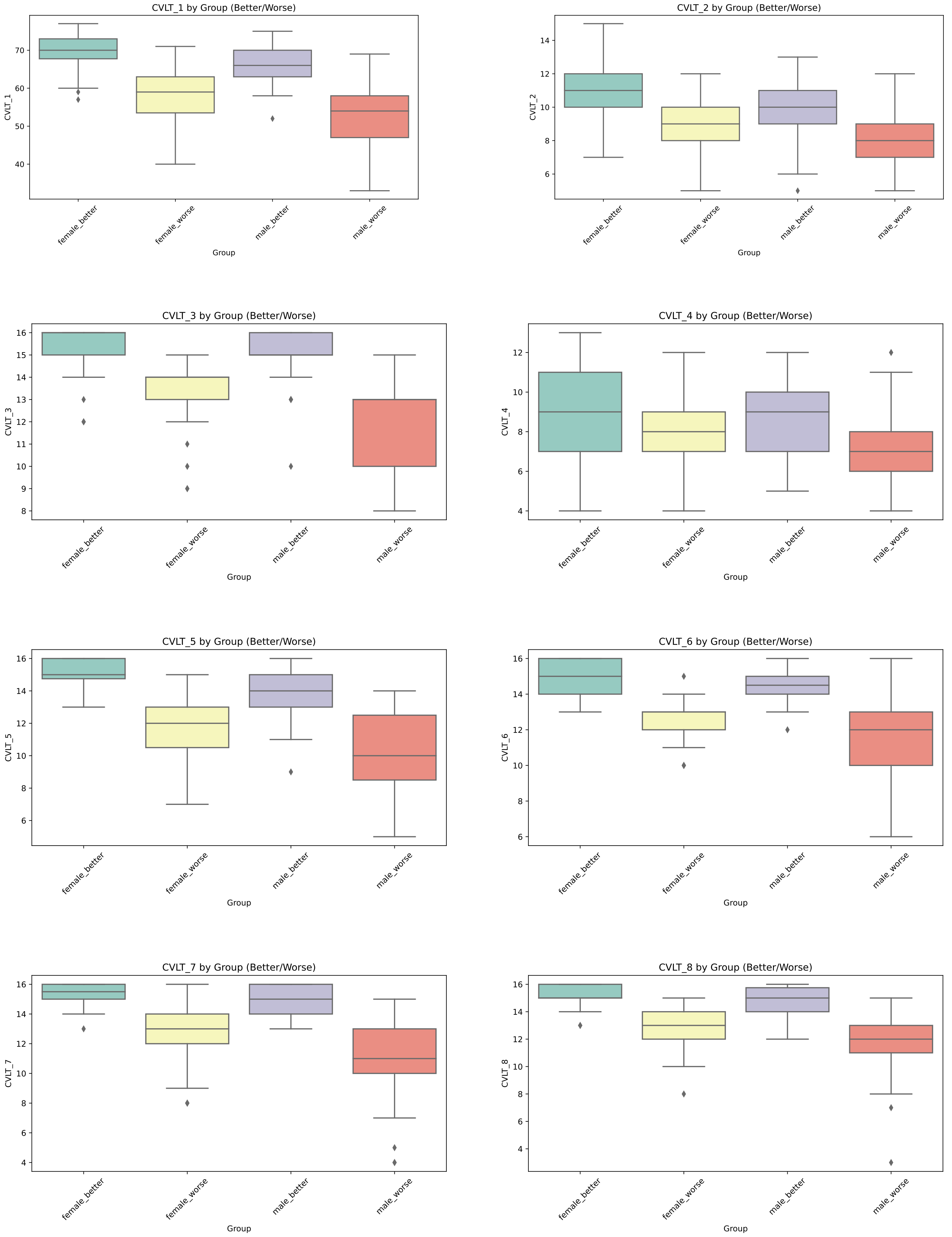

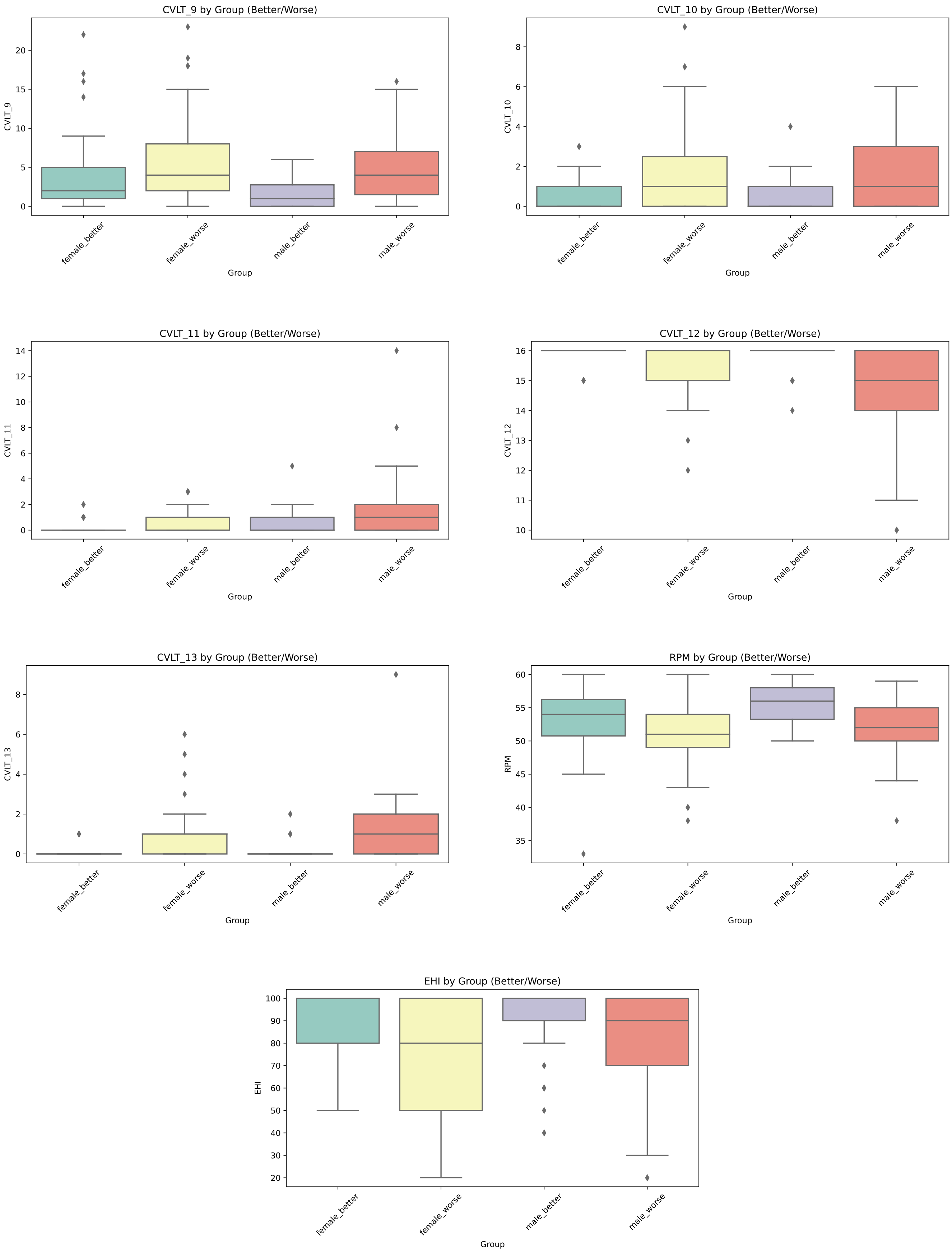

Figure S1Box plots of cluster comparisons, contrasting the better and worse cluster.

1.4 Statistical summary for non-cognitive variable

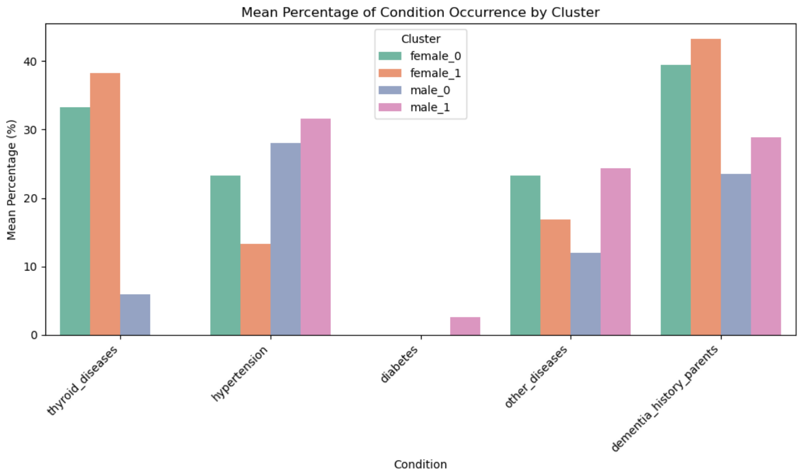

Figure S2Mean Percentage of Comorbidities by Cluster

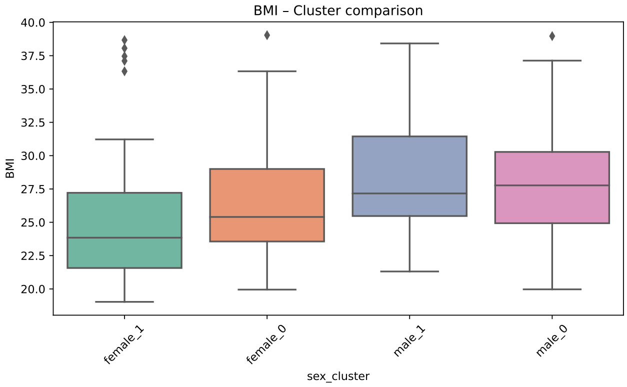

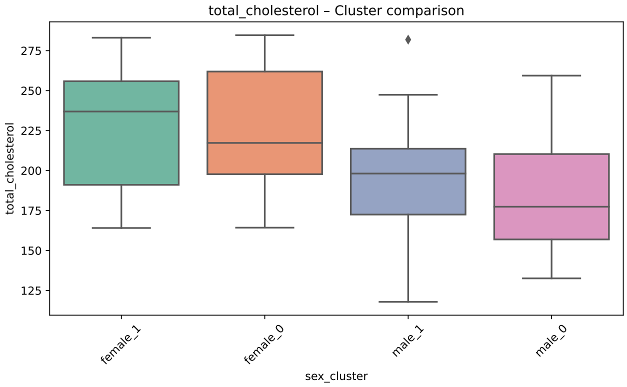

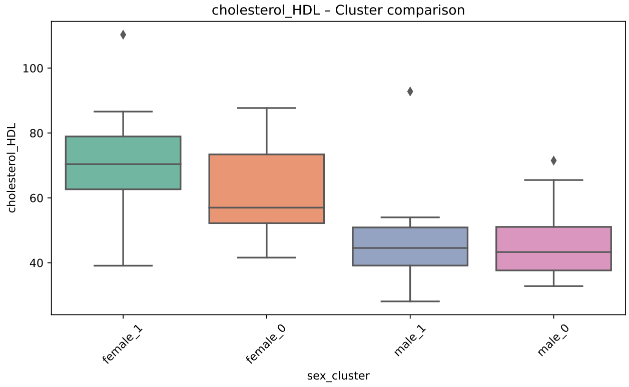

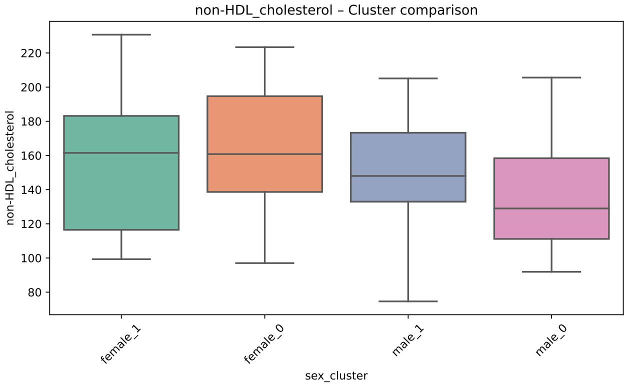

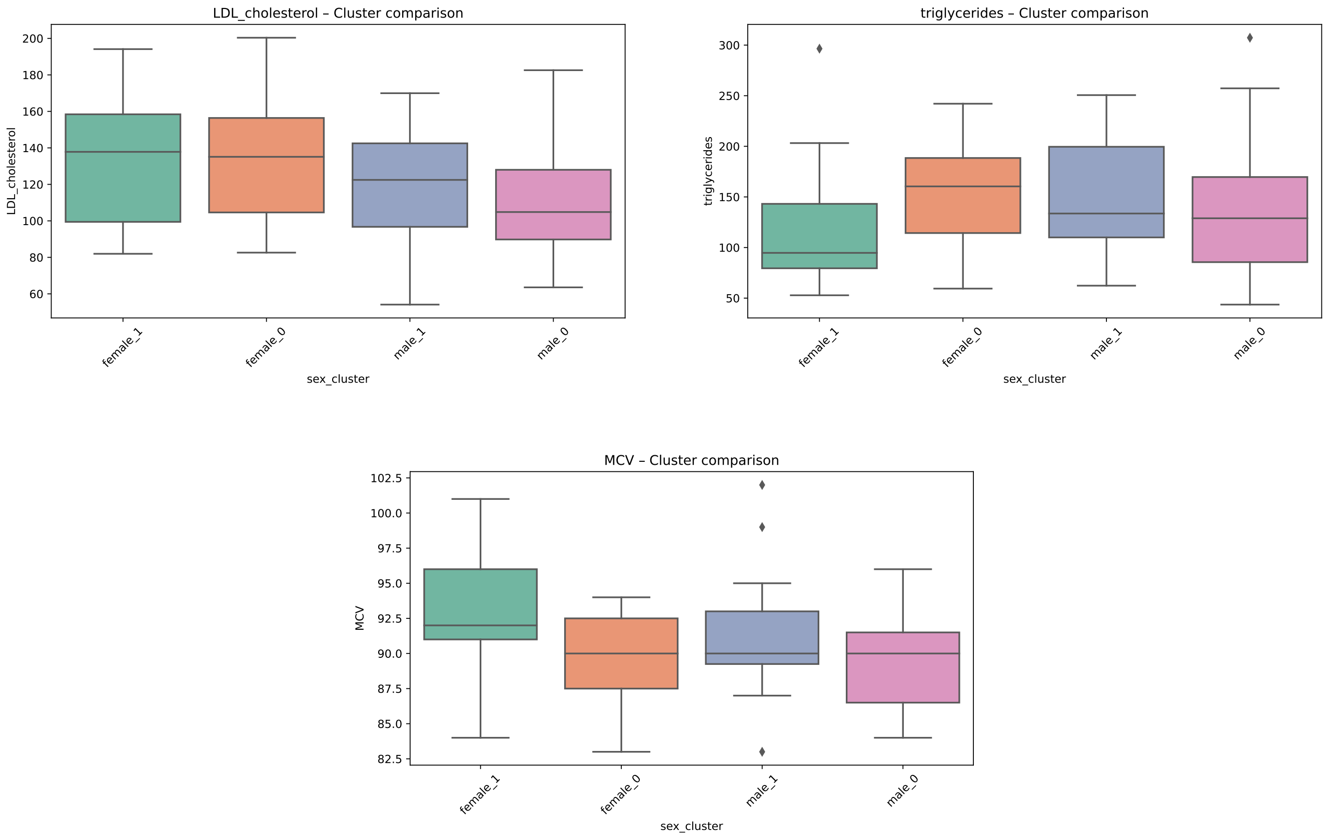

Figure S3Box plots of cluster comparisons for non-cognitive variables, contrasting the better and worse cluster within males and females.

Table S40. Summary statistics for non-cognitive variable variable, cluster and sex with ANOVA p-values

Variable	Cluster	Mean	SD	ANOVA p-value
BMI	female_0	26.58	4.31	0.0012
BMI	female_1	25.16	4.93	0.0012
BMI	male_0	28.10	4.13	0.0012
BMI	male_1	28.28	4.36	0.0012
total_cholesterol	female_0	226.13	41.02	0.0013
total_cholesterol	female_1	225.60	36.60	0.0013
total_cholesterol	male_0	184.95	36.14	0.0013
total_cholesterol	male_1	194.97	40.28	0.0013
cholesterol_HDL	female_0	62.28	14.95	0.0000
cholesterol_HDL	female_1	70.26	15.07	0.0000
cholesterol_HDL	male_0	46.57	11.40	0.0000
cholesterol_HDL	male_1	46.23	13.77	0.0000
non-HDL_cholesterol	female_0	163.85	37.84	0.2432
non-HDL_cholesterol	female_1	155.35	42.16	0.2432
non-HDL_cholesterol	male_0	138.37	33.14	0.2432
non-HDL_cholesterol	male_1	148.73	35.42	0.2432
LDL_cholesterol	female_0	133.99	35.42	0.1224
LDL_cholesterol	female_1	132.48	35.42	0.1224
LDL_cholesterol	male_0	110.57	34.51	0.1224
LDL_cholesterol	male_1	118.41	33.35	0.1224
triglycerides	female_0	149.27	52.80	0.1623
triglycerides	female_1	114.35	56.34	0.1623
triglycerides	male_0	139.02	68.80	0.1623
triglycerides	male_1	151.61	56.45	0.1623
MCV	female_0	89.33	3.70	0.0044
MCV	female_1	93.21	3.65	0.0044
MCV	male_0	89.47	3.32	0.0044
MCV	male_1	91.22	4.41	0.0044

1.5 Histograms of cognitive function variables

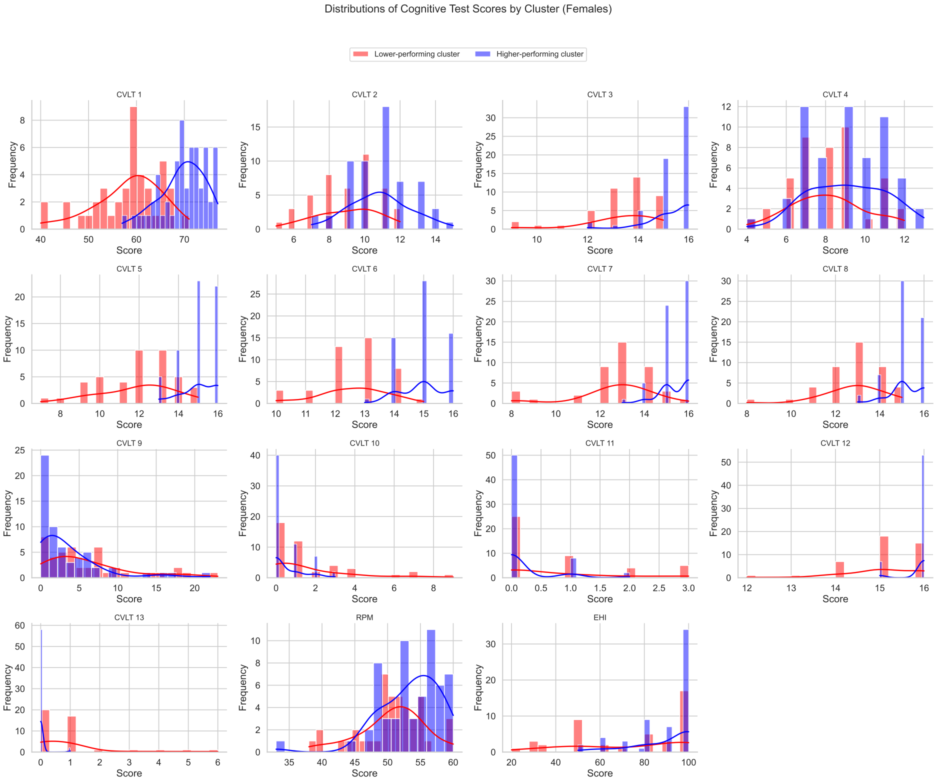

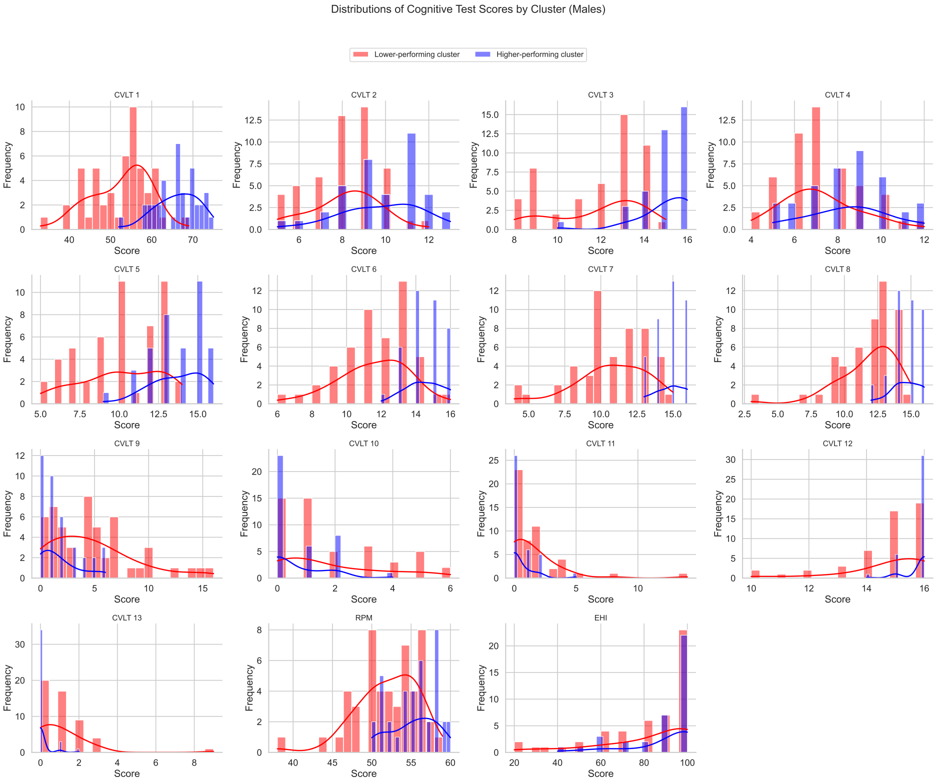

Figure S4 Histogram representation of cognitive functions variables comparing lower-performing, and higher-performing clusters.

1.6 Comparison of clustering

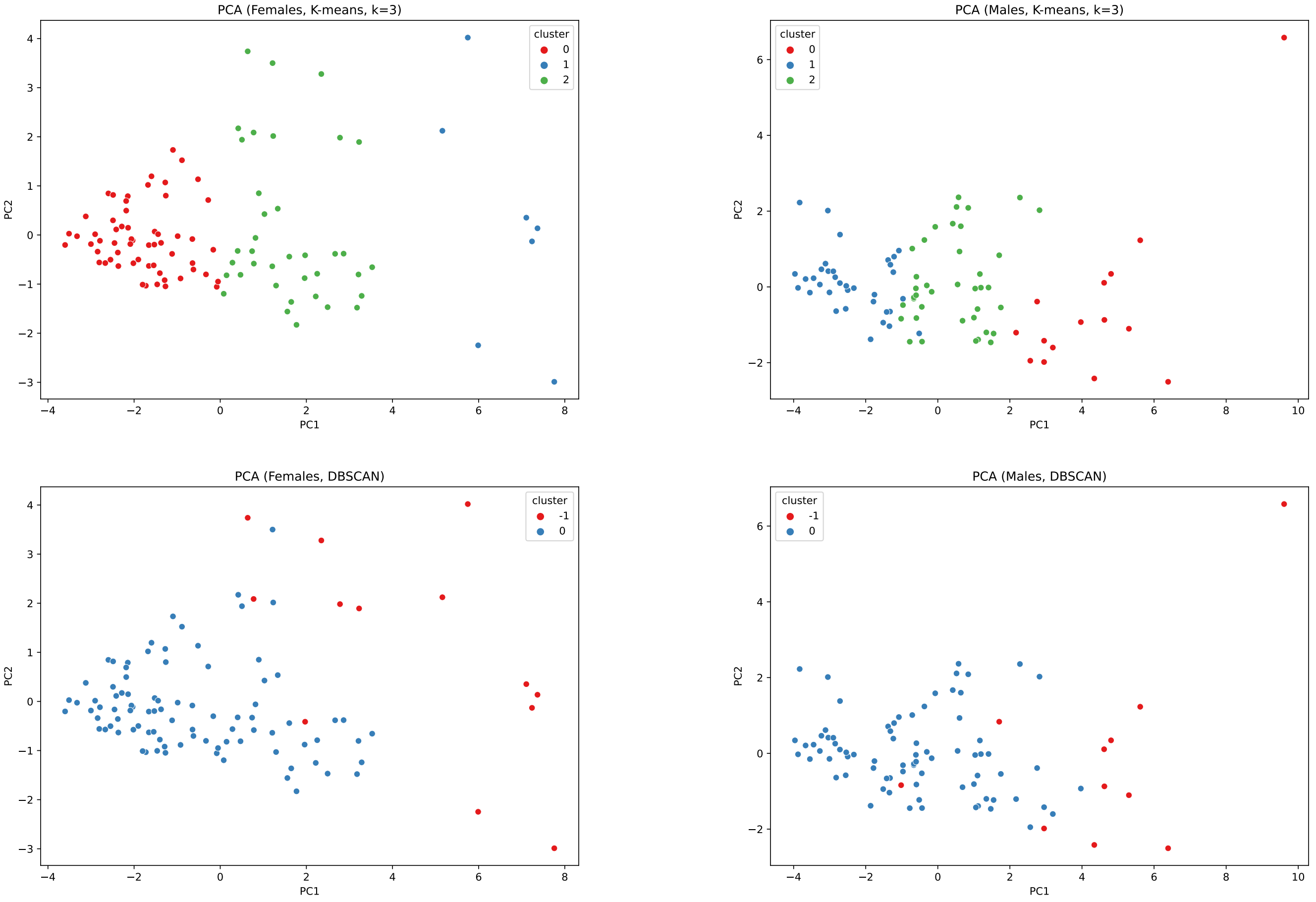

Figure S5 Clustering visualizations for K-Means and DBSCAN, shown for females (left panels) and males (right panels).
